# Impaired Airway Epithelial Barrier Integrity in Response to *Stenotrophomonas maltophilia* Proteases, Novel Insights Using Cystic Fibrosis Bronchial Epithelial Cell Secretomics

**DOI:** 10.3389/fimmu.2020.00198

**Published:** 2020-02-25

**Authors:** Kevin Molloy, Gerard Cagney, Eugene T. Dillon, Kieran Wynne, Catherine M. Greene, Noel G. McElvaney

**Affiliations:** ^1^Department of Medicine, Royal College of Surgeons in Ireland, Beaumont Hospital, Dublin, Ireland; ^2^School of Biomolecular and Biomedical Science, University College Dublin, Dublin, Ireland; ^3^Department of Clinical Microbiology, Royal College of Surgeons in Ireland, Beaumont Hospital, Dublin, Ireland

**Keywords:** *Stenotrophomonas maltophilia*, cystic fibrosis, extracellular proteases, secretomics, tight junction, epithelial barrier dysfunction

## Abstract

*Stenotrophomonas maltophilia* is a Gram-negative opportunistic pathogen that can chronically colonize the lungs of people with cystic fibrosis (CF) and is associated with lethal pulmonary hemorrhage in immunocompromised patients. Its secreted virulence factors include the extracellular serine proteases StmPR1, StmPR2, and StmPR3. To explore the impact of secreted virulence determinants on pulmonary mucosal defenses in CF, we examined the secretome of human CFBE41o- bronchial epithelial cells in response to treatment with *S. maltophilia* K279a cell culture supernatant (CS) using a liquid-chromatography-tandem mass spectrometry (LC-MS/MS) based label-free quantitative (LFQ) shotgun proteomics approach for global profiling of the cell secretome. Secretome analysis identified upregulated pathways mainly relating to biological adhesion and epithelial cell signaling in infection, whereas no specific pathways relating to the immune response were enriched. Further exploration of the potentially harmful effects of K279a CS on CF bronchial epithelial cells, demonstrated that K279a CS caused CFBE41o- cell condensation and detachment, reversible by the serine protease inhibitor PMSF. K279a CS also decreased trans-epithelial electrical resistance in CFBE41o- cell monolayers suggestive of disruption of tight junction complexes (TJC). This finding was corroborated by an observed increase in fluorescein isothiocyanate (FITC) dextran permeability and by demonstrating PMSF-sensitive degradation of the tight junction proteins ZO-1 and occludin, but not JAM-A or claudin-1. These observations demonstrating destruction of the CFBE41o- TJC provide a novel insight regarding the virulence of *S. maltophilia* and may explain the possible injurious effects of this bacterium on the CF bronchial epithelium and the pathogenic mechanism leading to lethal pulmonary hemorrhage.

## Introduction

Culture supernatant (CS) from bacteria, most notably *Pseudomonas aeruginosa*, has been used extensively to study host-pathogen interactions in the cystic fibrosis (CF) lung. Studies have explored its effects on TLR-induced inflammation (1), pro-inflammatory cytokine production (2, 3), innate immunity proteins (4–6) and degradation of extracellular matrix components (5, 7, 8), amongst others. However, there is a paucity of information regarding the effects of the important emerging CF pathogen, *Stenotrophomonas maltophilia* on airway epithelial cells *in vitro*. Given that the pathogenesis of *S. maltophilia* is complex and multifactorial, high-throughput technologies such as proteomics can help decipher differences in protein expression in composite circumstances such as host-pathogen interactions. Secretome analysis is a promising area of research permitting novel insights into the pathogenesis of different infections. Proteins secreted by a pathogen are present at the interface between the pathogen and the host cells and can thus regulate or mediate the host responses and cause disease (9).

The cell secretome is a collection of proteins that have been shed and proteins secreted by cells into the extracellular space and are important for maintaining cell-cell communication and proliferation. Examples of secretory proteins include extracellular matrix proteins, digestive enzymes, cytokines, chemokines, and growth factors (10). Identification of proteins released by cells into culture supernatants *in vitro* may help to better understand pathological conditions and mechanisms *in vivo*. For example, using high-throughput subcellular proteomics Lietzen et al. showed a robust secretion of different danger-associated molecular patterns in human macrophages in response to influenza A, and that the P2X7 receptor and Src tyrosine kinase activity are essential for inflammasome activation (11). Secretome analysis of A549 cells infected with *Mycoplasma pneumoniae* revealed higher levels of IL-33 mimicking *in vivo* conditions whereby higher than normal IL-33 levels are evident in plasma and bronchoalveolar lavage fluid from patients with *M. pneumonia*-associated pneumonia (12). Analysis of the *in vitro* proteome response of a human bronchial epithelial cell line to *Aspergillus fumigatus* demonstrated previously unknown aspects of bronchial epithelial cell behavior in response to infection including both cellular defense mechanisms and immune reactions (13).

Airway epithelial cells provide the first line of defense following exposure to inhaled infectious agents. Virulence factors such as secreted proteases expressed by *S. maltophilia* are likely to be important mediators of the pathogenic interaction between *S. maltophilia* and these cells. Indeed *S. maltophilia* has been shown to preferentially adhere along intercellular junctions, raising the possibility that tight junction dysfunction may be an important pathogenic mechanism of this bacterium (14). *S. maltophilia* has also been shown to induce morphological changes in fibroblast monolayers resulting in the cell layer partially condensing, formation of cell-free areas, and detachment from the culture plate (15). StmPR1 is likely a causative factor leading to the clinical observation of lethal pulmonary hemorrhage in those who are immunocompromised (16). Moreover, purified StmPr1 induces cell rounding and detachment of A549 cells by targeting cell integrin-extracellular matrix connections (matrilysis) as well as adherence and tight junction proteins for degradation (17, 18).

In this study, using K279a as the reference clinical strain for *S. maltophilia* infection, we provide an insight into host-pathogen interaction using a liquid-chromatography-tandem mass spectrometry (LC-MS/MS) based label-free quantitative shotgun proteomics approach for global profiling of the K279a CS treated human CFBE41o- (cystic fibrosis airway epithelial cell line) cell secretome. Using data from this secretomic analysis we examine the effects of K279a CS on epithelial barrier integrity and degradation of components of CFBE41o- cell tight junctions.

## Materials and Methods

### Reference Bacterial Strain

K279a was used as the reference clinical strain for this work (19). K279a was cultivated by scraping the surface of the frozen bacterial stock (−80°C) with a sterile 10 μL inoculating loop, placed in Luria-Bertani broth (LBB) and incubated overnight at 37°C on an orbital shaker at 200 rpm prior to use. Working stocks were maintained on agar plates at 4°C for up to 2 weeks. Cultures were regularly examined for purity using MALDI-TOF mass spectrometry (MS) analysis.

### Preparation of K279a Culture Supernatant (CS)

We have previously shown that Dulbecco's modified essential medium (DMEM) low glucose (5.6 mM) medium (Invitrogen) is the optimal growth medium for inducing K279a protease activity (20). To prepare a stock solution of K279a CS, 10 μL of an overnight K279a culture was inoculated in 6 x 15 mls of DMEM low glucose (5.6 mM) medium and grown for 48 h at 37°C on an orbital shaker. K279a CS was passed sequentially through 0.45-μm and 0.2-μm filters millex filters (Millipore Corporation, Bedford, MA). Culture supernatant (90 mls) was then concentrated using 5-kDa nominal-weight limit (NMWL) cut-off Amicon® Ultra-15 filter devices (Millipore Corporation, Bedford, MA). All concentrates were centrifuged at 4,000 × g and subsequently diafiltered by centrifugation with sterile DPBS to remove any low molecular weight contaminants including glucose and amino acids present in DMEM. An equivalent volume of DMEM was used as a negative control and for correction during protein quantification using the BCA (bicinchoninic acid) assay.

### Measurement of K279a CS Protease Activity

Protease activity was measured using the SensoLyte Red Protease Fluorometric Assay Kit (AnaSpec) as previously described (20). Prior to treatment of CFBE41o- airway epithelial cells, protease activity based on the known concentration of protein in K279a CS was adjusted based on a standard curve of K279a CS protease activity (measured in RFU/min).

### CFBE41o- Cell Culture

CFBE41o–, an SV40-transformed human ΔF508 homozygote bronchial epithelial cell line was maintained in 75 cm^2^ flasks at 37°C humidified CO_2_ incubator in minimal essential medium (MEM) supplemented with 10% fetal calf serum (FCS), 1% L-glutamine, 1% penicillin/streptomycin (Invitrogen). The cell line was originally obtained as a gift from D. Gruenert (California Pacific Medical Center Research Institute, San Francisco). Prior to treatment, cells were washed twice with sterile DPBS to remove excess FCS and were placed in serum free medium for 6 h. Immediately prior to treatment with K279a CS, cells were placed in fresh serum-free media.

### Romanowsky Stain of CFBE41o- Cells

To visualize morphological effects CFB41o- cells were untreated (control) or treated with K279a CS for 16 h at 37°C. Cells were stained using the Hema–Rapid staining set GURR® (VWR, UK), air dried and then fixed in methanol for 5 s following by staining with reagent one and reagent two for 3 and 6 s, respectively. Images were captured using an Olympus CKX41 and processed using CELL B by Soft Imaging System (Olympus, Tokyo, Japan).

### LDH CFBE41o- Cell Viability Assay

LDH assays were performed using the CytoTox 96® Non-Radioactive Cytotoxicity Assay (Promega, USA) according to the manufacturer's instructions. Absorbance was measured 490 nm on a microplate reader (Victor™ X3 Multilabel Plate Reader, PerkinElmer, Massachusetts, USA).

### CFBE41o- Cell Secretome In-Solution Digestion (ISD) and Mass Spectrometry

CFBE41o- cells were grown to 90% confluence, washed twice with warm DPBS and then either left untreated (control) or treated with K279a CS (protease activity: 500 RFU/min) for16 h. Supernatants were harvested, centrifuged at 4,500 × g for 10 min at 4°C and concentrated using Amicon-Ultra centrifugal filters (3-kDa NMWL) and protein concentration was determined by the BCA assay. Samples concentrations were adjusted to contain 20 μg of protein in 50 μL of 50 mM NH_4_HCO_3_ buffer for in solution digestion. Concentrated secretome samples were stored at −80° until processing for in solution digestion and mass spectrometry as described in the Supplementary Methods.

### Bioinformatic Data Analysis

Methods by which proteomic analysis, cellular localization of identified proteins, gene ontology analysis, prediction of disease related proteins using candidate gene prioritization and CFBE41o- cell secretome and sub-network analysis are described in the Supplementary Material.

### Tight Junction Studies

#### Monolayer Culture and TEER Measurement

CFBE41o- cells were seeded at 5 × 10^5^ cells/cm^2^ onto clear permeable filter inserts (Millipore Corporation, Bedford, MA, 6.5 mm diameter, 0.4 μm pore size), grown for 7 days prior to experiments, and medium was changed on alternate days. Prior to treatment, cells were washed with DPBS and the media replaced with serum free DMEM and allowed to equilibrate for 2 days prior to treatment. Tight junction integrity was assessed by measuring the TEER with an EVOM epithelial voltmeter in a Chopstick Electrode Set for EVOM (World Precision Instruments, Sarasota, FL, USA). The TEER of the background filter inserts was 20 Ω × cm^2^.

#### Fluorescein Isothiocyanate (FITC) Dextran Permeability Assay

Inserts were gently washed twice with 200 μL of Hanks' Balanced Salt Solution (HBSS), pre-warmed to 37°C then transferred using sterile tweezers, to a fresh 24 well plate. FITC-labeled dextran (10 kDa) was added (200 μL) to the upper chamber at a concentration of 0.5 mg/ml and 1 ml of pre-warmed HBSS added to the lower chamber. Cells were incubated for 1 h at 37°C. FITC dextran permeability was then measured by transferring 100 uL from each of the basal chambers to a black 96-well-plate and read using a microplate reader (Victor™ X3 Multilabel Plate Reader, PerkinElmer, Massachusetts, USA) at excitation 485 nm and emission 535 nm.

### Western Blotting

CFBE41o- cells were washed in ice cold DBPS and lysed for 15 min in 100 μL of RIPA buffer [50 mM Tris-Cl (pH 7.6), 1 mM EDTA (pH), 0.5 mM EGTA, 1% Triton X-100, 0.1% sodium deoxycholate, 0.1% SDS and 140 mM NaCl] in the presence of a protease inhibitor cocktail (Calbiochem, 539132), centrifuged at 14,000 × g for 5 min at 4°C and then heated to 95°C for 5 min prior to the addition of reducing sample buffer. Fifty microgram CFBE41o- lysate was separated using SDS-PAGE, transferred to PVDF using a semi-dry Novex XCell SureLock blotting system (ThermoFisher) and membranes were blocked (3% w/v Marvel skimmed milk, 1% bovine serum albumin in 0.1% PBS-Tween) then incubated with the respective primary antibodies for ZO-1 (1:500, rabbit polyclonal, ThermoFisher Scientific, #61-7300), Occludin (1:500, rabbit polyclonal, ThermoFisher Scientific, #71-1500), JAM-A (1:500, rabbit polyclonal, ThermoFisher Scientific, #36-1700), or Claudin-1 (1:500, rabbit polyclonal, ThermoFisher Scientific, #71-7800) overnight. β-actin was used a loading control (1:10,000 mouse monoclonal, Merck Millipore, #MAB1501). Visualization of immunoreactive protein bands was achieved using secondary antibodies to rabbit (Anti-rabbit IgG, HRP-linked antibody, 1:2,000, Cell Signaling, #7074S) or mouse (Anti-mouse IgG, HRP-linked antibody, 1:2,000, Cell Signaling, #7076S) and Immobilon Western chemiluminescent HRP substrate (Millipore) and the Syngene G:Box Chemi XL gel documentation system. Densitometry was performed using the GeneSnap Syngene program (Synoptics).

### Statistical Analysis

All statistical analyses were performed using GraphPad Prism 5.0 software package (San Diego, CA). All experiments were performed in triplicate and results are expressed as the mean ± SEM and were compared by Student's *t*-test (two-tailed) or analysis of variance where appropriate followed by Tukey *post-hoc* test for multiple comparisons where appropriate. Differences were considered significant at *p* ≤ 0.05.

## Results

### Morphological Effects of K279a CS on CFBE41o- Cells

**Figure 1:**
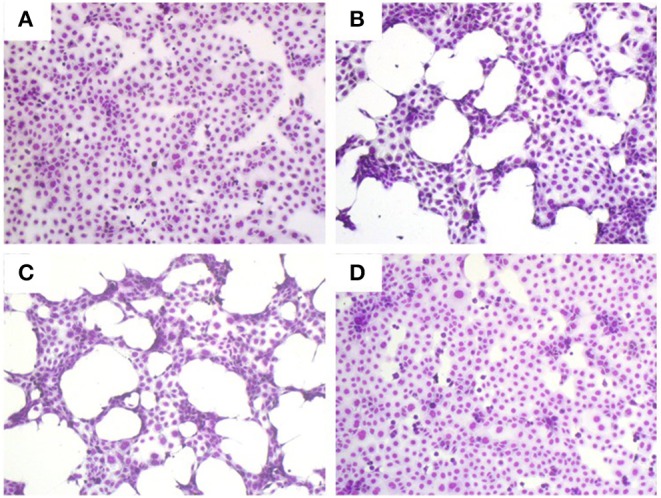
Romanowsky stain (Hema-Gurr) of untreated and treated CFBE41o- cells with K279a CS in the absence and presence of PMSF. Wells were seeded with CFBE41o- cells (3 × 10^5^ cells/ml) in MEM supplemented with 10% FCS and grown to 90% confluence in 24 well-tissue culture plates. The following day, medium was removed and washed twice with pre-warmed (37°C) DPBS to fully remove FCS. Cells were then placed in fresh serum free medium for 6 h prior to treatment. CFBE41o- cells were untreated (control, **A**) or incubated with 5% **(B)** or 10% v/v of K279a CS which was untreated **(C)** or treated with 1 mM PMSF **(D)** for 16 h. The following day the cell CS was removed and the remaining cells were stained using the Hema–Rapid staining set GURR® for hematology (VWR, UK). Cells were air dried and then fixed in methanol for 5 s following by staining with reagent one and reagent two for 3 and 6 s, respectively. Representative images were captured using an Olympus CKX41 and processed using CELL B by Soft Imaging System (Olympus, Tokyo, Japan).

There are many example reports using bacterial CS to study the behavior of virulence factors *in vitro* (3, 21–23). As a first step toward assessing the role, if any, of secreted proteases in the pathogenesis of *S. maltophilia* pulmonary infection, we examined the effect of differing concentrations of K279a CS (5 and 10% v/v) on CFBE41o- cell monolayers compared with untreated control cells (Figure 1A). Monolayers incubated with K279a CS displayed morphological changes including cell condensation, rounding and detachment after incubation for 16 h, the effects of which were most pronounced in those treated with 10% v/v K279a CS (Figures 1B,C). To determine whether extracellular serine protease(s) were responsible for these effects, K279a proteases were inhibited using 1 mM PMSF. The destructive effect of K279a CS was prevented by incubation with PMSF, which has been shown to be a potent inhibitor of K279a protease activity (Figure 1D).

### Label-Free Quantitative (LFQ) Shotgun Proteomic Analysis of CFBE41o- Secretome Following Treatment With K279a CS

To further elucidate the potential pathological changes occurring in cystic fibrosis airway epithelial cells following *S. maltophilia* infection, we used label-free quantitative (LFQ) shotgun proteomic analysis of the CFBE41o- secretome following treatment with K279a CS. We compared the relative abundance of proteins between two experimental conditions, CFBE41o- cells left untreated (control) and those treated with K279a CS (with protease activity of 500 RFU/min). Cells were treated for 16 h in the presence or absence of K279a CS followed by collection of cell secreted proteins (24). A schematic representation of the experimental design is outlined in Supplementary Figure 1.

LDH cytotoxicity assay showed that treatment with K279a CS did not significantly affect CFBE41o- cell integrity within 16 h. Cells treated with K279a CS with the highest protease activity (5 × 10^3^ RFU/min) released 40% greater LDH compared to control and the effect was prevented by PMSF. However, this was not statistically significant after correcting for multiple comparisons (Supplementary Figure 2).

**Figure 2:**
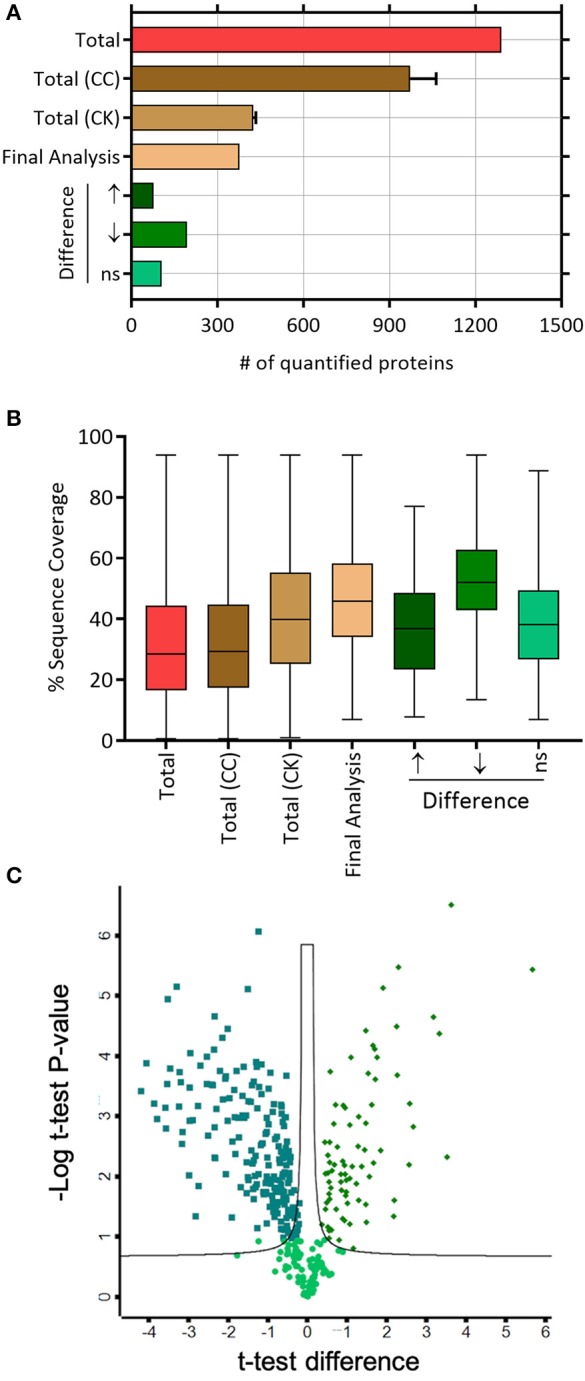
Label-free mass spectrometry secretome analyses of CFBE41o- cells treated with K279a CS. **(A)** Number of quantified proteins in the indicated protein fractions and experimental conditions. The mean and standard deviation are shown where necessary. **(B)** Box and whisker plots depicting the distribution of protein sequence coverage (coverage of tryptic peptides per protein in %). **(C)** Of the 376 proteins identified, statistically significant differentially expressed proteins (Benjamini Hochberg false discovery ratio 0.05) are shown in the volcano plot. Volcano plot analysis reveals that 271 proteins are significantly differentially expressed. Among the 271 proteins, 77 proteins were significantly elevated in CFBE41o- cells treated with K279a CS (green diamonds), whereas 194 proteins were significantly decreased (green squares). Abbreviations: CC, CFBE41o- cell control; CK, CFBE41o- cells treated with K279a CS.

**Figure 3:**
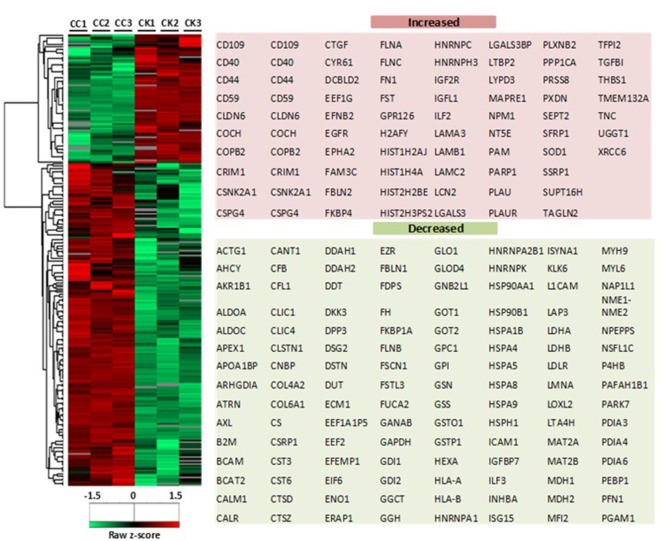
Heat map of CFBE41o- cell secretome in response to K279a CS. Heatmap and dendrograms were produced by unsupervised hierarchical clustering (Euclidean distance with complete linkage) of the indicated 271 significantly differentially expressed proteins (two sample *t*-test <0.05). The row Z-score or scaled expression value of each feature is plotted in red–green color scale. The red color of the tile indicates high abundance and green indicates low abundance. Gray color in the heatmap indicates “not detected.” The adjacent tables show the gene names of the proteins associated with the indicated clusters. CC, CFBE41o- cell control; CK, CFBE41o- cells treated with K279a CS.

Based on the LC-MS/MS data, 1290 proteins were identified, of which 972 ± 91 and 424.3 ± 9 were in the control group and the treatment group, respectively. A total of 376 proteins were included in the final analysis following filtration for proteins found in at least two out of three replicates in at least one group (Figure 2A). Among them, proteins were quantified on the basis of two or more peptides, with mean sequence coverage of 45.5 ± 17% (Figure 2B). Overall 271 statistically significant proteins were differentially expressed (Benjamini Hochberg false discovery rate <0.05) between treatment and control. Hierarchical clustering was also performed separately on the two groups (i.e., treated and untreated control), to determine proteins which were either up-regulated or down-regulated (Figure 3). Among those 271 proteins, 77 proteins were abundantly elevated in K279a CS treated cells, whereas 194 proteins were decreased (Figure 2C). A list of all identified and quantified proteins is presented in Supplementary Table 1.

### Characterization of Classically Secreted Proteins

**Figure 4:**
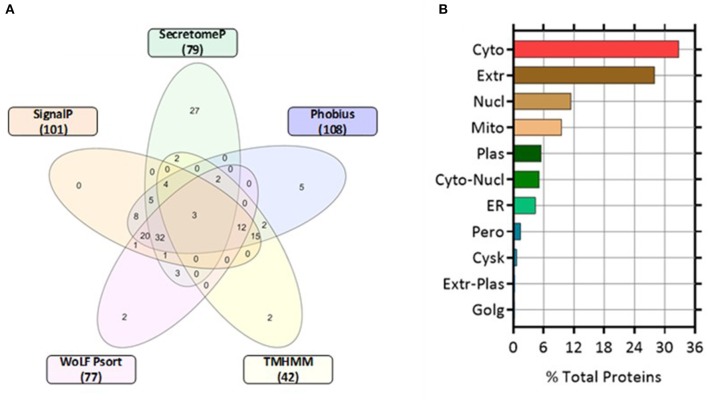
Five-Venn diagram and cellular locations of the identified proteins as predicted by bioinformatic analyses. The significant differentially expressed proteins (*n* = 271) were submitted to SignalP, Secretome P, Phobius, TMHMM, and WoLF Psort servers as outlined in the Materials and Methods section. Results from each program were compiled and the numbers of proteins predicted by each program individually were tabulated (Supplementary Table 1). **(A)** The numbers of proteins predicted by each program and all possible combinations are indicated in the Venn diagram **(B)** Location analysis of the differentially secreted proteins was based on WoLF Psort. Results are represented as percentage of the total number of analyzed proteins. Cyto, cytoplasm; Extr, extracellular; Mito, mitochondrion; Plas, plasma membrane; ER, endoplasmic reticulum; Cysk, cytoskeleton; Golg, Golgi apparatus; Nucl, nucleus; Pero, peroxisome.

Proteins classically secreted via an ER/Golgi dependent pathway normally have an amino-terminal secretion signal peptide sequence (25). We screened for both non-classically secreted (ER/Golgi independent pathway) proteins using SecretomeP and classically secreted proteins utilizing SignalP software. Of the 271 proteins, 79 were categorized as non-classical whereas 101 were designated as classically secreted. The shared agreement among the algorithms was good, with 52 entries fulfilling the set criteria for secretion through the classical pathway (SignalP prediction). Only 3 proteins were predicted to have a transmembrane (TM) domain while 98 proteins were not using SignalP-TM and SignalP-noTM to predict those proteins that might include TM regions. In contrast, TMHMM (a transmembrane helix prediction tool) predicted that 42 proteins had a TM domain. Phobius, a combined transmembrane topology and signal peptide predictor, predicted that 32 proteins contained both a signal peptide and a TM domain. This correlated well with combined SignalP and TMHMM analysis with 34 overlapping proteins predicted to have both a signal peptide and a TM. WoLF Psort was used to determine the subcellular location of the identified proteins, 77 proteins were determined as extracellular and represented 28% of the total number of quantified proteins. The remaining proteins were located in the cytoplasm (32.84%), nucleus (11.44%), mitochondrion (9.59%), plasma membrane (5.54%), and endoplasmic reticulum (ER, 4.43%) (Figure 4).

### Biological Significance of Identified Proteins

To understand the functional significance of the identified proteins, analyses of gene ontology for cellular component and biological function were classified using DAVID 6.8 (26). The analysis revealed major differences in the proteins that were differentially expressed in CFBE41o- cells treated with K279a CS compared to control. To enhance the power of disease-associated pathway detection, up-regulated and down-regulated proteins were analyzed separately [(27); Supplementary Figure 3]. Proteins residing in the extracellular region and extracellular matrix (ECM) were the most significantly altered in both groups but especially in the up-regulated proteins. Enrichment for cell junction was similar for both groups.

The terms biological adhesion, growth, and localization were enriched in the up-regulated proteins whereas cell killing, locomotion, and multi-organism process were enriched in down-regulated proteins. Notably, no significant differences in immune system processes were identified, indicating that the pathological response of CF bronchial epithelial cells in response to secreted *S. maltophilia* virulence factors may be structural rather than primarily immunostimulatory.

### Identification of Candidate Disease Proteins From PPI Networks Based on Identified Differentially Regulated Proteins

Protein-protein interaction (PPI) networks are critical to comprehensively understand cellular mechanisms and function. They have emerged as an important resource for understanding data from proteomics experiments in order to identify proteins which could play important pathogenic roles in lung disease (28). The importance of PPIs in disease pathogenesis was recently demonstrated in CF. A detailed analysis of the CFTR interactome identified key novel interactors whose loss promoted enhanced CFTR channel function indicating that global remodeling of ΔF508 CFTR interactions is crucial for CFTR rescue (29).

Network analysis may reflect the biological processes more objectively than analyzing individual proteins. The first step is to identify proteins of interest and these inputs (or “seed proteins”) are used to search for interactions from a curated PPI database. Constructing a PPI network (PPIN) using only the seed proteins may miss potentially important disease associated proteins and so completing the network with first order interactors by utilizing probabilistic PPINs improves the detection of candidate disease related proteins and disease pathways. This principle suggests that clues to the function of a protein can be obtained by seeing whether it interacts with another protein of known function (30). However, expanding a PPIN from a given set of “seed proteins” often leads to a complex PPIN lacking spatiotemporal consideration. To avoid this so-called “hairball” effect and to increase the robustness of our analysis we searched for shared candidate disease proteins among the top 100 interactors as ranked by three prioritization tools: NetworkAnalyst, GeneMANIA, and ToppGene.

In NetworkAnalyst, 161 up- and 348 down-regulated nodes were generated and the top 90 and 100 candidate proteins, respectively, were ranked. The interaction data from 100 related proteins generated using label propagation in GeneMANIA are summarized in Supplementary Table 2. Using ToppGene, 4,189 up- and 5,631 down-regulated first-order interactors of seed proteins were identified and ranked according to the network-based prioritization method (k-Step Markov, step size = 6) with a neighborhood distance of 1. The ranked list of the top 100 candidate proteins from the three prioritization tools are summarized in Supplementary Tables 3–5.

Overall comparison between the three networks identified 27 candidate proteins which were identified in two or more prioritization tools in both up-regulated and down-regulated networks. A list of these proteins and their basic information is described in Supplementary Table 6.

### Functional Classification of Identified Proteins

To better understand the nature of the identified proteins, KEGG pathway annotations were obtained. Eighteen pathways were significantly over-represented in the KEGG database (*P* < 0.01), 12 in the up-regulated and 6 in the down-regulated network. In the up-regulated proteins focal adhesion, various cancers, ECM-receptor interaction, EGFR/GRB2/KRAS (annotated here as dorso-ventral axis formation), and Gap and adherens junctions featured, as did pathways related to bacterial and parasitic infection (Supplementary Figure 4). In contrast, in the down-regulated protein network, pathways related to metabolism, protein processing in the ER and Parkinson's disease featured (Supplementary Figure 5).

### PPIN Module-Based Analysis Following Treatment of CFBE41o- Cells With K279a CS

Seed proteins and candidate proteins identified in both the up-regulated and down-regulated datasets were used for the construction of the final PPI network using STRING v10.0. Cluster analysis using the Reactome Functional Interaction (FI) app (Reactome FIViz) in Cytoscape v3.4.0 divided the up-regulated and down-regulated PPINs into five and seven modules, respectively (module 1 was excluded from further analysis secondary to an FDR > 0.01) (Supplementary Figures 6, 7). The most interesting terms are highlighted in bold; these relate to biological adhesion and bacterial infection. A summary of the over-represented KEGG pathways related to bacterial infection and their associated proteins can be found in Table 1 and a summary of the over-represented KEGG pathways related to biological adhesion and their associated proteins can be found in Table 2.

**Table 1:** Proteins identified from KEGG pathways related to bacterial infection.

**KEGG pathway**	**Network**	**Module**	**Proteins**^a^	
			**Seed**	**Candidate**
Ep. cell signaling in *H. pylori* infection	↑	2	EGFR	SRC, TJP1
Legionellosis	↓	4	HSPA8, VCP	
Pathogenic *E. coli* infection	↓	5	ACTG1, EZR, TUBA1B	ACTB
		7	YWHAZ	YWHAQ
Shigellosis	↓	5	ACTG1, VCL, PFN1	ACTB
*Salmonella* infection	↓	5	ACTG1, MYH9, PFN1	ACTB
Bacterial invasion of epithelial cells	↓	5	ACTG1, VCL	ACTB

a *Proteins divided by (1) Seed proteins: identified from LFQ (label-free quantification) shotgun proteomics analysis and (2) Candidate proteins: Candidate disease proteins identified using prioritization tools*. *Ep, Epithelial; ↑, up-regulated network; ↓, down-regulated network*.

**Table 2:** Proteins identified from KEGG pathways related to biological adhesion.

**KEGG Pathway**	**Network**	**Module(s)**	**Proteins^a^**	
			**Seed**	**Candidate**
Focal adhesion	↑	2, 3, 5	COL4A2, EGFR, FLNA, FN1, LAMA3, LAMB1, LAMC2, PPP1CA, TNC, THBS1	COL1A1, CAV1, GRB2, LAMA5, SRC
Gap junction	↑	2	EGFR	GRB2, KRAS, SRC, TJP1
Tight junction	↑	2	CLDN6	KRAS, SRC, TJP1
	↓	5	ACTG1, MYH9, SPTAN1	ACTB
Adherens junction	↑	2	EGFR	SRC, TJP1
	↓	5	ACTG1, VCL	ACTB
ECM-receptor interaction	↑	5	AGRN, LAMA3, LAMB1, LAMC2	LAMA5
	↓	6	COL4A2, COL6A1, FN1	
ECM organization	↓	6	COL4A2, COL6A1, CTSD, EFEMP1, FBLN1, FN1, SERPINE1, SPARC, TIMP1	

a *Proteins divided by (1) Seed proteins: identified from LFQ (label-free quantification) shotgun proteomics analysis and (2) Candidate proteins: Candidate disease proteins identified using prioritization tools*. *↑, up-regulated network; ↓, down-regulated network*.

### Epithelial Barrier Integrity in CFBE41o- Cells Is Disrupted by Secreted K279a Serine Proteases

Using secretomics, we found that pathways related to biological adhesion and extracellular matrix components were significantly enriched following treatment with K279a CS. To extrapolate this further we sought to determine if serine proteases secreted by K279a had an effect on attachment of the CFBE41o- epithelial monolayer. Airway epithelial barrier function was determined by measuring TEER. In initial experiments, various concentrations of K279a CS with differing protease activity were added to the apical surface of CFBE41o- monolayers seeded in transwell permeable supports. Using repeated measures of analysis to evaluate the effect of K279a CS on TEER, apical treatment of CFBE41o- cell monolayers with K279a CS (protease activity of 5 × 10^3^ RFU/min) significantly decreased monolayer resistance by 24 h (*p* < 0.002). The observed drop in TEER was protease mediated given the abrogation of the observed effects by PMSF (*p* = 0.007) (data not shown).

**Figure 5:**
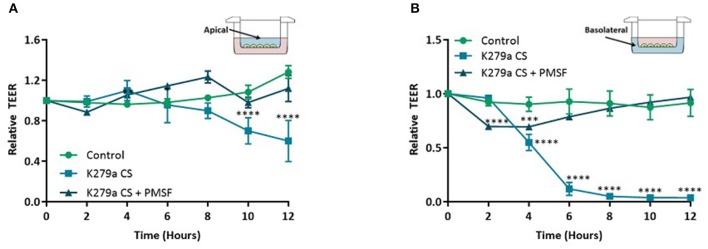
TEER in CFBE41o- monolayers following treatment with K279a CS. CFBE41o- cells were seeded at a density of 5 × 10^5^ cells/cm^2^ onto clear permeable filter inserts (6.5 mm diameter, 0.4 μm pore size). Cells were grown for 7 days in MEM supplemented with 10% FCS and medium was changed on alternate days. Prior to treatment, cells were washed with DPBS to remove any residual FCS and the media replaced with serum free medium (SFM) and allowed to equilibrate for a further 48 h. The day before treatment cells were placed in fresh SFM overnight. CFBE41o- cell monolayers were left untreated (control) or treated with K279a CS (CS) (protease activity = 5 × 10^3^ RFU/min) in the absence or presence of PMSF (1 mM). Transepithelial electrical resistance (TEER) was measured using an EVOM epithelial voltmeter in an Endohm-6 chamber. To construct TEER curves, measurements were taken every 2 h. **(A)** TEER curve of apically treated cells. **(B)** TEER curve of basolaterally treated cells. All results are representative of three independent experiments. Results are expressed as relative TEER to time zero (T0). Treatment vs. control: ****p ≤ 0.0001, ***p ≤ 0.001; Two-way-ANOVA followed by Tukey *post-hoc* test for multiple comparisons.

Further analysis of TEER kinetic curves demonstrated that addition of K279a CS to the apical surface of TEER monolayers resulted in a time-dependent decrease in monolayer resistance. TEER was significantly lower at 10 h in cells apically treated with K279a CS compared with controls cells (*p* < 0.0001) which indicated disruption of epithelial barrier integrity, the effect of which was prevented by PMSF (*p* = 0.0004). At 12 h, TEER was 60.1 ± 11.75% relative to the control (*p* < 0.0001; Figure 5A). In comparison, addition of K279a CS to the basolateral surface of CFBE41o- cell monolayers resulted in a significantly more rapid time-dependent decrease in monolayer resistance. After 4 h, cells treated with K279a CS had a lower TEER compared with controls (*p* < 0.0001). At 12 h, TEER was only 3.62 ± 0.09% relative to the control (*p* < 0.0001; Figure 5B). As observed in apically treated cells, this effect was abrogated in the presence of PMSF, which indicated that secreted serine protease(s) were responsible for this effect.

### K279a CS Increase Paracellular Permeability to Macromolecular FITC-Dextran

**Figure 6:**
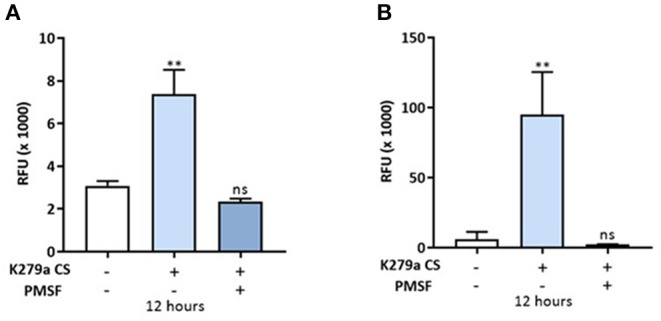
*In vitro* permeability assay to FITC-dextran in CFBE41o- monolayers treated with K279a CS. FITC-dextran permeability (RFU × 1,000) in CFBE41o- cell monolayers was assessed in apically and basolaterally treated compartments after 12 h following treatment with K279a proteases (5 × 10^3^ RFU/min) in the absence and presence of PMSF. Increased permeability to FITC dextran was observed in apically treated cells **(A)** but the effect was more pronounced when monolayers were treated basolaterally **(B)**. All measurements are means ± SEM from biological replicates. Treatment vs. control: **p ≤ 0.01, one-way ANOVA followed by Tukey *post-hoc* test for multiple comparisons.

We next assessed the permeability of K279a CS (protease activity = 5 × 10^3^ RFU/min) treated CFBE41o- monolayers with the macromolecular tracer, FITC-dextran (10 kDa), which can only transverse the monolayer via the paracellular route. At 12 h, when the development of TEER was significantly reduced in both apically and basolaterally treated cells, the permeability of FITC-dextran was measured. Apically treated monolayers displayed a 2.393 ± 0.2108-fold increase in permeability to FITC-dextran compared with control (*p* = 0.009) whereas a 15.54 ± 2.882-fold increase was observed in basolaterally treated monolayers (*p* = 0.002). In both instances, the permeability of FITC-dextran was prevented by PMSF, linking K279a protease activity to increased paracellular macromolecular transport (Figure 6).

### Secreted K279a CS Degrade the Tight Junction Proteins ZO-1 and Occludin

Given that we observed a decline in TEER and increase in paracellular macromolecular permeability in CFBE41o- cell monolayers following treatment with K279a CS, we further investigated the integrity of tight junction proteins. Secretome analysis of CFBE41o- cells treated with K279a CS showed that the tight junction protein claudin-6 was up-regulated and that TJP1 or ZO-1 were identified as candidate disease associated proteins by PPIN analysis. We chose to examine the expression of ZO-1, occludin, JAM-A and claudin-1. Claudins 1, 3, 4, 5, 7, 8, and 18 are expressed in human bronchi and bronchioles whereas claudin-6 expression may be an effect of immortalization of the CFBE41o- epithelial cell line as it has been reported in non-small cell lung cancer (31) and in developing lung tissue (32). Therefore, we focussed on claudin-1 rather than claudin-6 as the expression of former is more constitutive in the adult lung epithelium.

**Figure 7:**
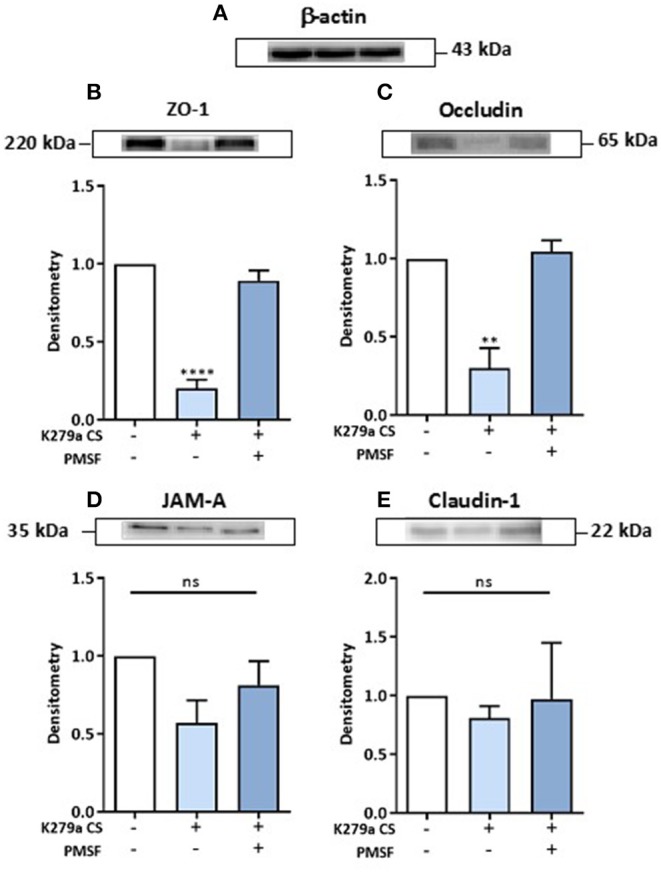
Effect of K279a CS on the tight junction proteins: ZO-1, occludin, JAM-A and claudin-1. CFBE41o- cells were seeded at a density of 1 × 10^6^ cells/ml onto 100 mm Triple Vented Tissue Culture Dishes. Cells were grown to 90% confluence in MEM supplemented with 10% FCS and medium was changed on alternate days. Prior to treatment, cells were washed with DPBS to remove any residual FCS and the media replaced with serum free medium (SFM) and allowed to equilibrate overnight. On the day of treatment, cells were placed in fresh SFM and treated for 4 h. Cells were left untreated (control) or treated with K279a proteases (5 × 10^3^ RFU/min) in the absence or presence of PMSF (1 mM). Western blots were performed on whole-cell lysates (50 μg of protein) from CFBE41o- cells probed with antibodies to β-actin **(A)**, ZO-1 **(B)**, occludin **(C)**, JAM-A **(D)**, and claudin-1 **(E)**. Histograms represent the densitometric fold change in relative protein expression (relative to untreated controls) in response to treatment following correction with β-actin. All results (expressed as relative densitometry units) are representative of three independent experiments. All measurements are means ± SEM from biological replicates. ****P < 0.001, **P ≤ 0.01; one-way-ANOVA with Tukey correction for multiple comparisons.

CFBE41o- cells were either untreated (negative control) or treated with K279a CS (protease activity 5 × 10^3^ RFU/min) in the presence or absence of PMSF (1 mM) for 4 h. Following normalization to negative controls and correction to β-actin (Figure 7A), ZO-1 (*p* < 0.0001) and occludin (*p* = 0.003) were significantly degraded, effects that were prevented in the presence of PMSF (Figures 7B,C). No significant changes for JAM-A or claudin-1 were observed (Figures 7D,E). Therefore, we concluded that extracellular serine proteases from *S. maltophilia* degrade the tight junction proteins ZO-1 and occludin, but not JAM-A or claudin-1.

## Discussion

In this study, we analyzed the proteins secreted by CFBE41o- cells in response to treatment with *S. maltophilia* K279a CS. In total we identified 77 proteins which were significantly up-regulated and 194 proteins which were significantly down-regulated in response to treatment. Gene ontology and pathway analysis demonstrated that biological adhesion and assembly of extracellular matrix components were significantly enriched terms within the up-regulated proteins whereas cellular metabolism was over-represented in the down-regulated proteins. Using the seed proteins identified from this study we identified candidate disease proteins to determine the effect of *S. maltophilia* on CF bronchial epithelial cells using PPIN analysis from three prioritization tools: NetworkAnalyst, GeneMANIA, and ToppGene. Using modular analysis of the PPIN generated from the seed proteins and candidate disease proteins we identified six modules related to bacterial infection and five modules related to biological adhesion.

*Stenotrophomonas maltophilia* has been shown to preferentially adhere along intercellular junctions, raising the possibility that tight junction dysfunction may be an important pathogenic mechanism of this bacterium (14). In our analysis epithelial cell signaling related to *Helicobacter pylori* infection was significantly over-represented in the up-regulated PPIN. *H. pylori*, a spiral, gram negative rod plays an important role in the pathogenesis of peptic ulcer disease and like *S. maltophilia*, it preferentially attaches to cell-cell interfaces (33). *H. pylori* can alter the function of the apical junctional complex resulting in changes of structure, function and morphology of gastric epithelial cells. Translocation of the protein CagA into these cells induces recruitment of the tight junction proteins ZO-1 and JAM to the sites of bacterial attachment and may serve to target and retain *H. pylori* at epithelial cell-cell junctions (34). In addition to alterations in tight junction assembly, *H. pylori* can alter expression of tight junction proteins. ZO-1 expression is decreased whereas claudin-4 is increased in *H. pylori* infected individuals indicating that damage to the gastric epithelial barrier function may be important in the pathogenesis of *H. pylori* peptic ulcer disease (35).

*Stenotrophomonas maltophilia* has been shown to induce morphological changes in fibroblast monolayers resulting in the cell layer partially condensing, formation of cell-free areas, and finally detachment from the culture plate (15). StmPR1 is a likely factor responsible for the clinical observation of lethal pulmonary hemorrhage in those who are immunocompromised (16). Although loss of structural components are important factors negatively affecting epithelial integrity, loss of function of key protease inhibitors and an increase in endogenous protease activity may also be relevant. TIMP1 and TIMP2, natural inhibitors of matrix metalloproteinases were down-regulated in response to K279a CS. We identified MMP2 (matrix metalloproteinase-2), a type IV collagenase as a candidate disease protein in the up-regulated protein network. *P. aeruginosa* has been shown to increase MMP-2 activity in CFBE41o- cells and a gain of functional MMP-2 and loss of function of TIMPs 1 and 2 are possible causes of epithelial damage in *S. maltophilia* lung disease (23). Other important anti-proteases which were down-regulated included alpha-1 antitrypsin and plasminogen activator inhibitor (PAI-1), an inhibitor of fibrinolysis, the absence of which predisposes the individual to a haemorrhagic diathesis. This is likely to have an important role in the pathogenesis of lung disease as *S. maltophilia* degrades the innate immune proteins: alpha-1 antitrypsin, secretory leukoprotease inhibitor and elafin (20).

In addition, the observed over enrichment of biological pathways involving biological adhesion indicated a possible causative role for secreted *S. maltophilia* proteases in disassembly of intercellular (tight, adherens, and gap) junctions. The cardinal work by Windhorst et al., examining the extracellular protease profile of *S. maltophilia* showed that the extracellular protease StmPR1 had significant pathological effects on fibroblasts and these effects were abrogated in the presence of the StmPR1 inhibitor chymostatin (15). Our observations using TEER measurements of CFBE41o- cell monolayers exposed to K279a CS are consistent with the suggested role of secreted extracellular proteases having a role in the pathogenesis of CF lung disease. Our conclusion is supported by several lines of evidence. Firstly, we demonstrated a significant disruption of the epithelial monolayer with morphological changes in cultured CFBE41o- cells which was abrogated in the presence of the protease inhibitor PMSF. Secondly, the TEER of CFBE41o- cells, which is higher than their non-CF counterparts, 16HBE14o- (36), was significantly reduced following treatment with K279a CS in comparison to the untreated control. Thirdly, the tight junction proteins ZO-1 and occludin but not JAM-A or claudin-1 were degraded in CFBE41o- cells following treatment with K279a CS, the effect of which was prevented by PMSF. The importance of this deleterious effect in the pathogenesis of CF has been demonstrated in other well-known CF pathogens, and disruption of epithelial barrier integrity may be one of the mechanisms inducing chronic inflammation in cystic fibrosis, similar to that observed in inflammatory bowel disease (37).

Like *S. maltophilia*, live *P. aeruginosa* is also capable of disrupting tight junctions in apically treated VA10 monolayers within 24 h of infection as measured by a gradual drop in TEER and a concomitant decrease in the expression of the tight junction protein, ZO-1 (22). *P. aeruginosa* have been shown to invade airway epithelial barriers by destroying tight junctions (38), while *Pseudomonas* elastase can disrupt the tight junction in human nasal epithelial cells by downregulating the transmembrane proteins claudin-1 and-4, occludin, and tricellulin (39). ER stress induced by *P. aeruginosa* has also been implicated as a cause of tight junction destruction in primary bronchial epithelial cells (40). More recently, a strong correlation between *in vitro* elastase activity of clinical isolates of *P. aeruginosa* and mucosal barrier dysfunction has been demonstrated. These changes were seen in conjunction with degradation of ZO-1, occludin and β-actin and implicate *P. aeruginosa* exoproteins in the pathophysiology of *P. aeruginosa* associated chronic rhinosinusitis by severely compromising mucosal barrier structure and function (41).

Others have shown similar effects using xps mutants of K279a, a key regulatory gene of the type II secretory system from *S. maltophilia*. DuMont et al. demonstrated rounding, detachment, and death of A549 cells, an adenocarcinoma human alveolar epithelial cell line, mediated via degradation of ECM components such as type I collagen and fibronectin by the major and minor extracellular proteases StmPr1 and StmPr2 (17, 42). More recently, we and others have identified an intermediate protease, StmPR3, as an additional potential virulence factor of *S. maltophilia* (18, 20). Interestingly, StmPr3 showed xps-mediated rounding and detachment of A549 cells, as well as xps-mediated degradation of fibronectin, fibrinogen, and interleukin-8 (IL-8), similar to StmPR1 and StmPR2 (18). Additionally, purified StmPR1 has been shown to degrade the tight junction protein occludin and the basolaterally expressed adherens junction protein E-cadherin. The observed ability of StmPR1 to degrade E-cadherin within 1 h of co-incubation may in part explain the relative increased speeds of TEER reduction seen here following treatment of the basolateral compartment with K279a CS compared to the apical one (18). From a clinical perspective, continued exposure of the immunocompetent host epithelium to *S. maltophilia* proteases may induce microbleeds in the lung such as in the context of CF. However, in the immunocompromised host the inability of the host to control the virulence of the pathogen could permit access to the basolateral compartment of the pulmonary epithelium leading to pulmonary hemorrhage.

The ability of extracellular serine proteases from *S. maltophilia* to denude the airway epithelium likely confers a growth advantage for the bacterium. K279a and other clinical isolates of *S. maltophilia* have siderophore-like activity when grown at 37 °C in low-iron media and a mutation in one of the predicted biosynthesis genes (entC) impairs the production of the siderophore and reduces bacterial growth in low-iron conditions (43). The ability of *S. maltophilia* to cause microbleeds within the CF lung likely has beneficial effects for growth of the organism *in vivo* but harmful effects for the host. Degradation of host iron-containing proteins by neutrophil elastase in the CF lung is a source of iron (44) that can promote growth of *S. maltophilia* via FecA mediated transport of exogenous siderophore ferric citrate from the environment into the bacterial periplasm (45). Moreover, release of haem can be harmful to the CF patient as haem can stimulate IL-8 from CFBE41o- cells (46).

This study has a number of limitations. Firstly, K279a, the reference clinical strain of *S. maltophilia* used in this work was isolated from the blood of a cancer patient and its virulence determinants may differ from CF strains of the bacterium (47). However, there is considerable overlap in the major extracellular protease gene (StmPR1) between CF *S. maltophilia* isolates and K279a with 70% of CF-derived strains carrying the 1,621-bp allelic variant of StmPr1 present in the K279a reference genome (48). Secondly, we did not specifically examine the ability of endogenous antiproteases [e.g., alpha-1 antitrypsin (AAT)] to abrogate the effect of extracellular proteases in K279a CS on epithelial barrier integrity. Thirdly, by maintaining CFBE41o- cell monolayers in a submerged culture rather than at an air-liquid interface there may have been a dilutional effect which may have reduced the inhibitory ability of endogenous antiproteases on K279a CS. Alpha-1 antitrypsin is an endogenous inhibitor of neutrophil elastase with an extracellular pulmonary epithelial concentration ~10% that of serum levels (49). While production of AAT and other antiproteases are normal in CF, the neutrophil elastase burden is so large that it overwhelms the normal anti–neutrophil elastase protection (50). We have recently shown that K279a CS is capable of degrading the endogenous proteases inhibitors AAT, SLPI, and elafin (20) and thus chronic colonization with *S. maltophilia* is an additional combatant to overwhelm the anti-protease armory within the CF lung. The use of aerosolised AAT is an attractive therapeutic option. Not only could it inhibit NE mediated IL-8, TNF-α, and LTB4 production (50), but it may also potentially inhibit extracellular bacterial proteases including those from *S. maltophilia*.

In conclusion, using secretomics we have provided a unique insight into the pathogenesis of *S. maltophilia* in CF lung disease. Using this data we have shown that one of the primary pathogenic mechanisms in *S. maltophilia* infection involves disruption of epithelial barrier integrity. We confirmed this by demonstrating a time dependent reduction in TEER and an increase in paracellular permeability, an effect mediated by degradation of the tight junction proteins ZO-1 and occludin. Future work to examine the relative contribution of purified StmPR1, StmPR2, and StmPR3 and mutant K279a strains lacking the aforementioned proteases will shed further light on the pathogenic potential of this emerging multi-drug resistant CF pathogen.

## Data Availability Statement

The datasets generated for this study are available on request to the corresponding author.

## Author Contributions

KM, CG, GC, ED, and NM contributed conception and design of the study. KM, GC, ED, and KW contributed to mass spectrometic analysis. KM performed the experiments, organized the data, performed the statistical analysis, and wrote the first draft of the manuscript. All authors contributed to manuscript revision, read, and approved the submitted version.

### Conflict of Interest

The authors declare that the research was conducted in the absence of any commercial or financial relationships that could be construed as a potential conflict of interest.
