# Supplementary material for: Impaired Airway Epithelial Barrier Integrity in Response to Stenotrophomonas maltophilia Proteases, Novel Insights Using Cystic Fibrosis Bronchial Epithelial Cell Secretomics
Source: Front Immunol. 2020 Feb 25;11:198. doi: 10.3389/fimmu.2020.00198 (PMC7053507; doi:10.3389/fimmu.2020.00198)
Supplement: Supplementary file 1 [file Data_Sheet_1.docx]

**SUPPLEMENTARY MATERIAL**

**SUPPLEMENTARY METHODS**

**CFBE41o- cell secretome in-solution digestion (ISD) and mass spectrometry**

***In-solution digestion (ISD) of CFBE41o- cell secretome***

ISD of the protein samples was performed in preparation for mass spectrometry. Protein concentrations were adjusted with 50 mM NH_4_HCO_3_ buffer to 20 µg in 50 µl as previously described (Molloy et al., 2019). Proteins were solubilised and denatured in the presence of 5.5 µl of 1 % w/v RapiGestTM solution resulting in a final concentration of 0.1% w/v RapiGestTM and denatured on at heating block at 80°C for 10 minutes. Samples were subsequently reduced in the presence of 3.7 µl 80 mM TCEP (tris2-carboxyethylphosphine) at 60°C for 60 min (final TCEP concentration of 5 mM). The samples were alkylated in the dark in the presence of 3-fold molar excess (15 mM) of iodoacetic acid (IAA) by adding 4.8 µl 200 mM iodoacetic acid (IAA). Trypsin was prepared from proteomics grade Trypsin Singles (Sigma, T7575) by adding 1 μL of Trypsin Solubilization Reagent to each of the Trypsin Singles vials followed by 49 μl of the prepared Trypsin Reaction Buffer. Prepared trypsin (25 μL containing 0.5 μg of trypsin) was added to the sample to digest the proteins. Samples were incubated in an air circulation thermostat and samples were incubated overnight at 37°C with agitation at 350 rpm. The following day the digestion was stopped and precipitated, by adding 8.9 µl of 10% v/v formic acid to a final concentration of 1% v/v. The sample was incubated for 30 minutes at 37°C with agitation at 350 rpm. The sample was briefly vortexed and subsequently centrifuged at 16,000 x g for 10 minutes and the supernatant was harvested. The extracts were dried in a vacuum centrifuge at 60°C for 30 minutes and resuspended in 20 μL of sample preparation solution (0.5% trifluoracetic acid, TFA) for ZipTip Sample Preparation.

***Zip-Tipping sample preparation***

The sample was aspirated and dispensed through a ZipTip (Millipore) to bind, wash, and elute the peptides. Each ZipTip was wetted by aspirating 10 µl of 50 % acetonitrile in 0.1 % TFA (wetting and elution solution) into the tip and slowly dispensing it out onto tissue. A further 10 µl of 0.1 % TFA in ddH_2_0 (equilibration and washing solution) was aspirated and dispensed onto tissue. This step was repeated. For peptide binding, the sample was aspirated up and down ten times inside the sample tube. Peptides bound within the tip were subsequently washed by aspirating and dispensing 10 µl of equilibration and washing solution. Finally, for releasing the peptides from the ZipTip, 10 µl of wetting and elution solution was aspirated up and down three times and released in a new sample tube. Sample tubes were then placed in a vacuum centrifuge and solvent was evaporated. Samples were resuspended in 20 μL of washing solution and frozen at -20°C prior to injection on LC/MS/MS.

***LC-MS/MS Analysis***

LC-MS/MS analysis following in-solution digestion was carried out on a Thermo Scientific Q Exactive mass spectrometer connected to a Dionex Ultimate 3000 (RSLCnano) chromatography system as previously described (Molloy et al., 2019).

**Bioinformatic data analysis**

***Proteomic analysis***

Further analysis of the MaxQuant-processed data was performed using the in-house developed Perseus software (version 1.5.3.2). The “proteingroups.txt” file produced by MaxQuant was loaded into Perseus. Firstly, protein groups consisting of only identified by site proteins, reverse hits and contaminants were removed. Then the label-free quantification (LFQ) intensities were logarithmized and each sample was assigned into a corresponding group (CC=control and CK=K279a treated cells). Proteins not found in at least two out of three replicates in at least one group were omitted from the analysis. A two-sided t-test with 250 randomizations was performed between the treatment and the control. A significance threshold was determined at an FDR of 0.05 and s0 = 0.2 on the post imputed dataset to identify statistically significant differentially secreted proteins. In order to identify up-regulated and down-regulated proteins in treated and untreated samples, hierarchal clustering analysis was performed on the significantly differentially secreted proteins by z-score normalisation prior to analysis and the Euclidean distance was selected as a similarity measure.

***Cellular localisation of identified proteins***

Classically secreted proteins were screened for by utilising SignalP (version 4.1) software (http://www.cbs.dtu.dk/services/SignalP) (Petersen et al., 2011). Subsequently all proteins lacking the classical signal peptide for the translocation to the ER, i.e. the so-called non-classical secretory pathways (e.g. through transporters or exosomes) were then tested by SecretomeP (version 2.0) software (http://www.cbs.dtu.dk/services/SecretomeP/) (Bendtsen et al., 2004). Signal peptides for secretion were also analysed using Phobius (http://phobius.cbr.su.se/) (Kall et al., 2004). Using the TMHMM algorithm (http://www.cbs.dtu.dk/services/TMHMM/) (Sonnhammer et al., 1998), prediction of transmembrane α-helices was based on the expected number of amino acid residues in transmembrane helices (ExpAA). An ExpAA number greater than 18 was used to denote if a protein was likely to be transmembrane. Additionally, WoLF PSORT (http://www.genscript.com/wolf-psort.html ) (Horton et al., 2007) was interrogated for predicting the sub-cellular location of proteins. Overlaps between the five cellular localisation tools were visualised using InteractiVenn. InteractiVenn (http://www.interactivenn.net) is a web-based tool to examine set unions in Venn diagrams of sets of biological elements, affording a flexible approach to observations on the interactions between joined sets (Heberle et al., 2015).

***Gene ontology (GO) annotation of identified proteins***

Analysis of gene ontology (GO) of classification was performed using the ‘‘Database for annotation, visualization, and integrated discovery’’ (DAVID) (DAVID Bioinformatics Resources 6.8. National Institute of Allergy and Infectious Diseases (NIAID), NIH, Bethesda, MD (Huang da et al., 2009). GO analysis was performed for up-regulated and down-regulated proteins using ontologies to systemically describe the identified proteins based on biological function and cellular compartment.

***Prediction of disease related proteins using candidate gene prioritisation***

A large number of tools are available for candidate gene/protein prioritisation, but they use different data sources and as such data on protein-protein interactions (PPIs) are highly heterogeneous. No single PPI network (PPIN) database can capture all relevant relations. Combining multiple molecular network approaches has been asserted as a promising tool to discover new immune targets in diseases (Yang et al., 2016). To this end, we utilised three different data sources (NetworkAnalyst, GeneMANIa and ToppGene) to identify target proteins in the PPI network based on the assumption that similar phenotypes are caused by genes/proteins with similar or related functions.

NetworkAnalyst (http://www.networkanalyst.ca) is a web-based tool designed to perform various common and complex meta-analyses of gene expression data via a web interface. The NetworkAnalyst uses a comprehensive high-quality PPI database downloaded from the InnateDB. InnateDB, a member of the International Molecular Exchange (IMEx) consortium, was created by manually curating protein interaction data from published literature as well as from the Molecular Interaction (MINT) database; the IntAct database; the Database of Interacting Proteins (DIP); the General Repository for Interaction Datasets (BioGRID)and the Biomolecular Interaction Network Database (BIND) (Xia et al., 2014). Using their Uniprot accessing numbers, up-regulated and down-regulated proteins were mapped to the PPI network database (InnateDB) to generate a protein-protein interactome.

GeneMANIA (http://www.genemania.org) is a flexible, user-friendly web interface for analyzing gene lists and prioritizing genes for functional assays. Data sets are collected from publicly available databases, including co-expression data from Gene Expression Omnibus (GEO); physical and genetic interaction data from BioGRID and predicted protein interaction data based on orthology from I2D. Pathway and molecular interaction data are obtained from Pathway Commons, which contains data from BioGRID, Memorial Sloan-Kettering Cancer Center, Human Protein Reference Database, HumanCyc, Systems Biology Center New York, IntAct, MINT, NCI-Nature Pathway Interaction Database and Reactome (Warde-Farley et al., 2010). GeneMANIA uses the ‘guilt-by-association’ function prediction approach which hypothesizes that clues to the function of a protein can be obtained by seeing whether it interacts with another protein of known function (Oliver, 2000). A ‘seed list’ of known related genes is submitted and this is then extended to include other genes that are predicted to share a similar function based on overlapping connection within the biological networks. Default advanced setting were used in GeneMANIA, and the query gene sets including HGNC gene symbols of up-regulated and down-regulated proteins were submitted as the input. The top 100 related genes were obtained using at most 20 attributes using automatic weighting as the final result.

ToppGene ((http://toppgene.cchmc.org) is a functional annotation-based disease candidate gene prioritization tool that uses a fuzzy-based similarity measure for gene list functional enrichment and for the identification and prioritization of novel disease candidate genes in the interactome. ToppGene performs better than SUSPECTS, PROSPECTR and ENDEAVOUR, frequently used methods in candidate gene prioritization. In this study, top ranked candidate genes were identified in the interactome based on both functional annotations and PPIN analysis using the ToppGeNet function (Chen et al., 2009). Seed lists containing Entrez IDs of the up-regulated and down-regulated proteins were submitted as the training set and first order interactors (proteins with a seed distance of 1) were obtained to generate a test set on the basis of network prioritization method. The K-Step Markov prioritization method was selected, and step size was set at 6. The top 100 ranked genes in the test set were obtained according to their prioritization score.

InteractiVenn was used to analyse the top 100 (n = 90, Network Analyst) ranked proteins identified from the three data sources. Proteins which intersected two or more data sources were identified as the most promising candidate proteins. Circos plots (http://circos.ca/) (Krzywinski et al., 2009) illustrated the relationship between the differentially identified protein dataset, the candidate disease proteins and significant KEGG (Kyoto Encyclopedia of Genes and Genomes) (Kanehisa et al., 2016) pathways (p<0.05) identified using NetworkAnalyst.

***CFBE41o- cell interactome and sub-network analysis***

The upregulated and downregulated datasets and identified disease-related candidate protein targets were used as seed terms for the construction of the final PPI network using STRING (Search Tool for the Retrieval of Interacting Genes, version 10.0). The STRING database is dedicated to functional associations between proteins and generates both physical and functional PPIs from various sources including known experimental interactions, pathway knowledge parsed from manually curated databases as well as interactions predicted de novo by a number of algorithms using genomic information and also using co-expression analysis (Szklarczyk et al., 2015). The PPIN was constructed from the list using a high confidence score of 0.7.

The PPIN was visualized using Cytoscape network visualization software version 3.4.0 and analyzed using the Network Analyzer plug-in. The Reactome Functional Interaction (FI) app (Reactome FIViz) was used to construct network modules using spectral partition based network clustering (Newman, 2006). Modules were annotated using the ‘Analyze module function’ to enrich biological pathways. To allow better interpretation of biological pathways, the use of network modules allows the detection of patterns occurring significantly more often than occur beyond random expectation in the complex networks. Only pathways that had an FDR below 0.01 were included in the final KEGG pathway analysis.

**SUPPLEMENTARY FIGURES**

**Supplementary Figure 1.** Schematic workflow of the proteomic protocol for the identification and quantification of the proteins secreted by CFBE41o- cells in response to K279a culture supernatant.

**Supplementary Figure 2. Effect of K279a culture supernatant on CFBE41o- cell cytotoxicity and epithelial attachment.** Wells were seeded with CFBE41o- cells (2x10^5^ cells/well) in MEM supplemented with 10% FCS and were grown to overnight confluence in a 96 well tissue culture plate. The following day, medium was removed and washed twice with pre-warmed (37°C) DPBS. Cells were then placed in fresh serum free medium for 6 hours prior to treatment. CFBE41o- cells were either untreated (negative control) or treated with increasing concentrations of K279a culture supernatant (CS) (protease activities = 1.25, 2.5 and 5 × 10^3^ RFU/min) +/- PMSF (1 mM) for 16 hours. A negative control for PMSF (1 mM) was included as well as positive controls including whole cell lysis buffer treatment of control cells and LDH supplied in the CytoTox 96® Non-Radioactive Cytotoxicity kit. Absorbance was measured at 490 nm on a microplate reader. % Cytotoxicty was calculated as: 100 × [(Experimental LDH Release (OD_490_)/ Maximum LDH Release (OD_490_). All results are representative of three independent experiments. Data is reported as means ± SEM from biological replicates. One-way-ANOVA followed by Tukey post hoc test for multiple comparisons: ****(p≤0.001), Untreated controls and K279a CS vs LDH control; ** (p≤0.01) Untreated controls and K279a CS vs whole cell lysis control; ns (non-significant), K279a CS (5 × 10^3^ RFU/min) vs K279a CS + PMSF.


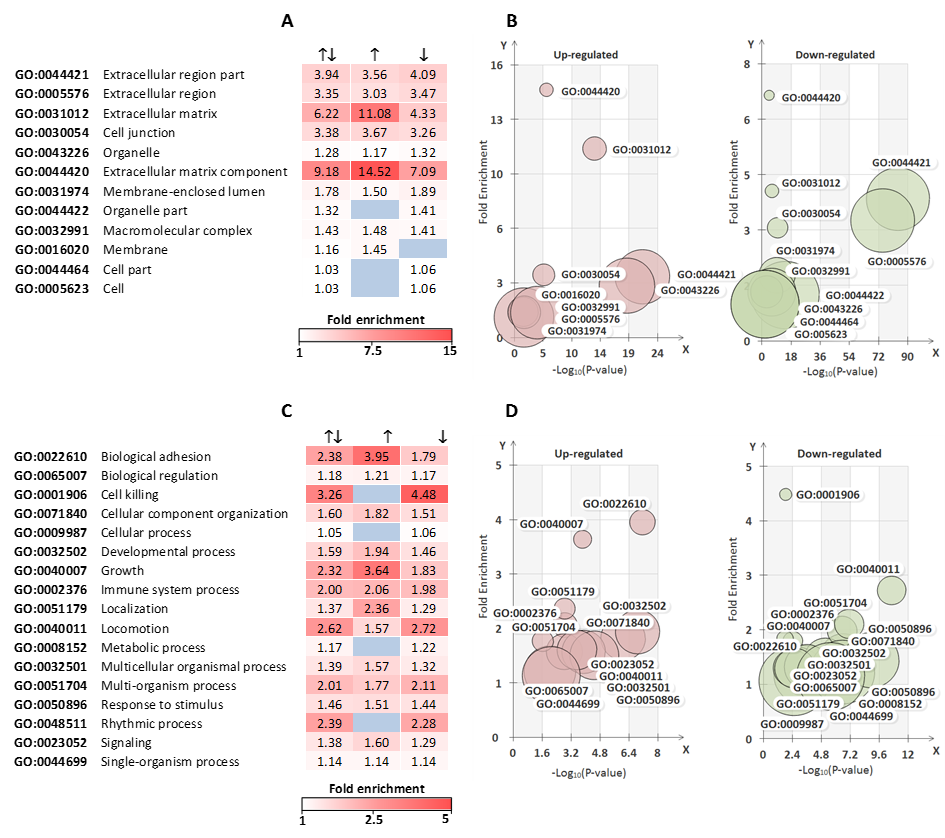


**Supplementary Figure 3. Heatmaps and bubble plots of highly significant gene ontology (GO) annotations of identified proteins.** Using DAVID 6.8, GO annotations for cellular component (top) and biological process (bottom) were found to be increased or decreased by K279a CS compared to control. Panel (A+C) Heatmaps illustrating the significantly enriched GO terms in up-regulated (↑), down-regulated (↓) and in all proteins (↑↓). Panel (B+D) Bubble plots were used to illustrate GO terms in up-regulated (left) and down-regulated (right) in response to treatment. Fold enrichment is assigned to the y-axis and the negative logarithm of the adjusted Benjamini p-value to the x-axis and bubble size indicates the identified number of proteins per GO term. Over-representation statistics were calculated by using the hypergeometric analysis and Benjamini & Hochberg False Discovery Rate (FDR) correction (p<0.05).


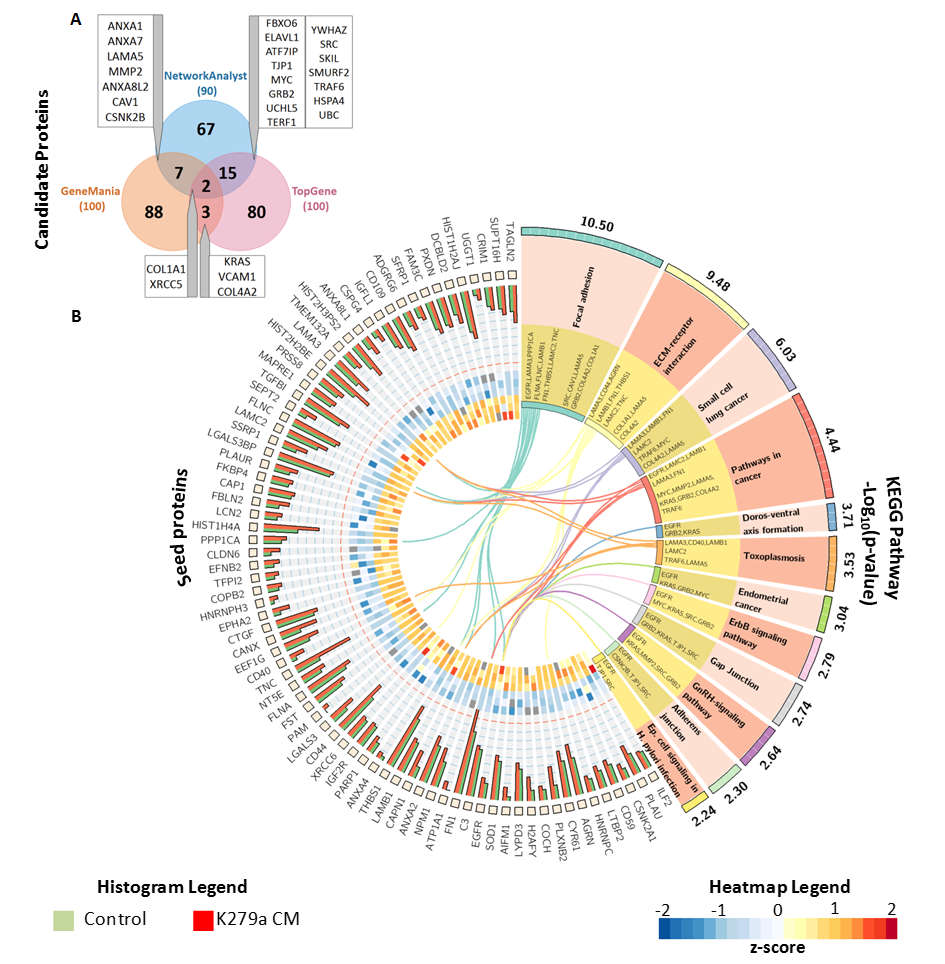
**Supplementary Figure 4. CIRCOS plot based on the KEGG pathway analysis of the up-regulated protein network data.** (A) Venn diagram: Shows the candidate proteins co-existing between NetworkAnalyst, GeneMANIA and ToppGene. (B) Circos Plot: The histogram illustrates the Log10 LFQ (label-free quantification) intensity of identified proteins between K279a treated (red) and untreated (green) CFBE41o- cells. The heat map shows z-scores of identified proteins between K279a treated (inner circle) and untreated (outer circle) CFBE41o- cells. The curved lines in the center represent the linkage between the seed proteins identified by MS analysis and their associated KEGG pathways. KEGG pathways are denoted by alternating segments (right side) and the related proteins are listed in the inner part of the annotated segments (yellow).
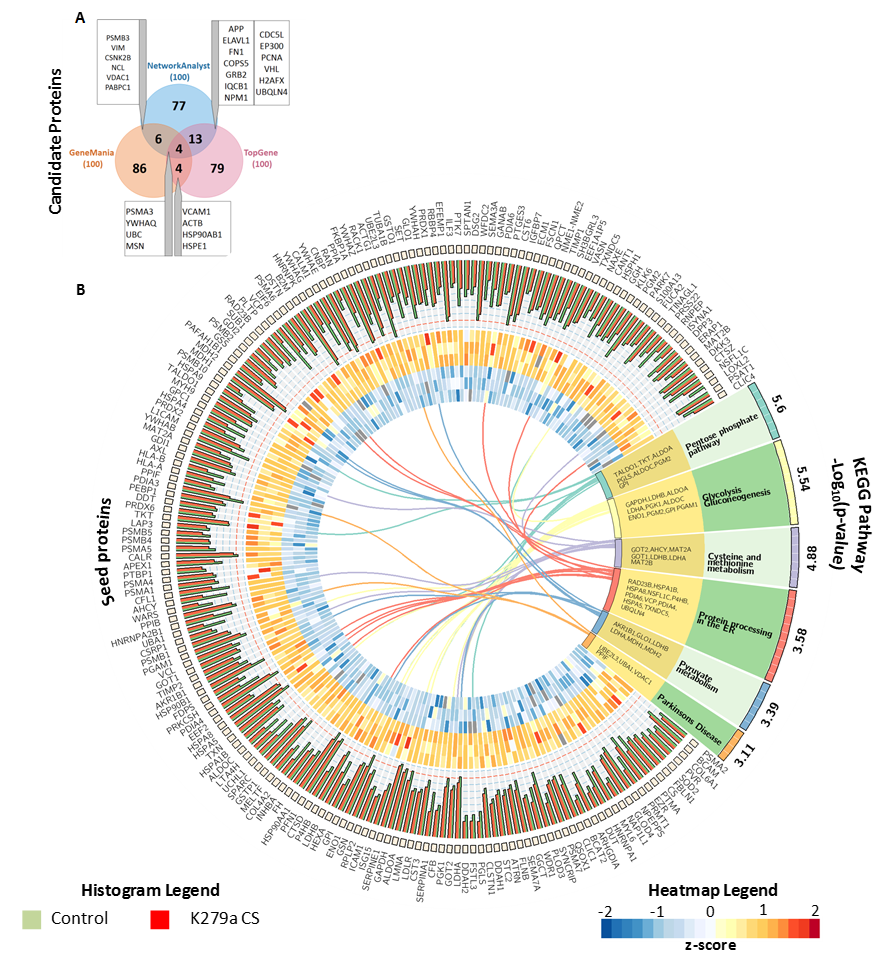


**Supplementary Figure 5. CIRCOS plot based on the KEGG pathway analysis of the down-regulated protein network data.** (A) Venn diagram: Shows the candidate proteins co-existing between NetworkAnalyst, GeneMANIA and ToppGene. (B) Circos Plot: The histogram illustrates the Log10 LFQ (label-free quantification) intensity of identified proteins between K279a treated (red) and untreated (green) CFBE41o- cells. The heat map shows z-scores of identified proteins between K279a treated (inner circle) and untreated (outer circle) CFBE41o- cells. The curved lines in the center represent the linkage between the seed proteins identified by MS analysis and their associated KEGG pathways. KEGG pathways are denoted by alternating segments (right side) and the related proteins are listed in the inner part of the annotated segments (yellow).


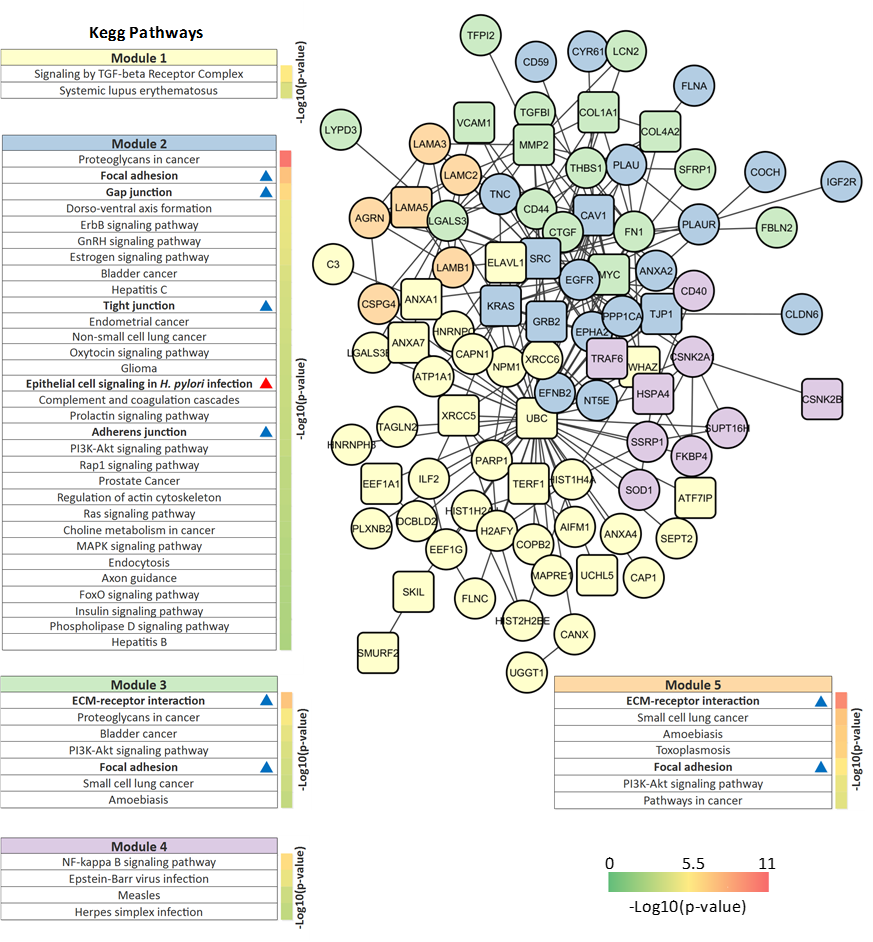


**Supplementary Figure 6. A protein–protein interaction network (PPIN) for the up-regulated proteins in K279a CS treated CFBE41o- cells.** The PPI network was generated using STRING v10.0. Each module was created using the Reactome FIViz app in Cytoscape v3.4.0 and each was assigned a unique colour. Seed proteins are indicated by circles and non-seed proteins by squares. Modules were analysed using the ‘Analyze module function’ to enrich biological pathways. Only pathways that had a false detection rate (FDR) below 0.01 were included in the final KEGG pathway analysis. Annotated pathways and the weight of their associated -Log10(p-value) are highlighted next to their respective pathway as denoted by the –Log10(p-value) color key. KEGG pathways relating to bacterial infection are indicated by a red triangle and those relating to biological adhesion by a blue triangle.


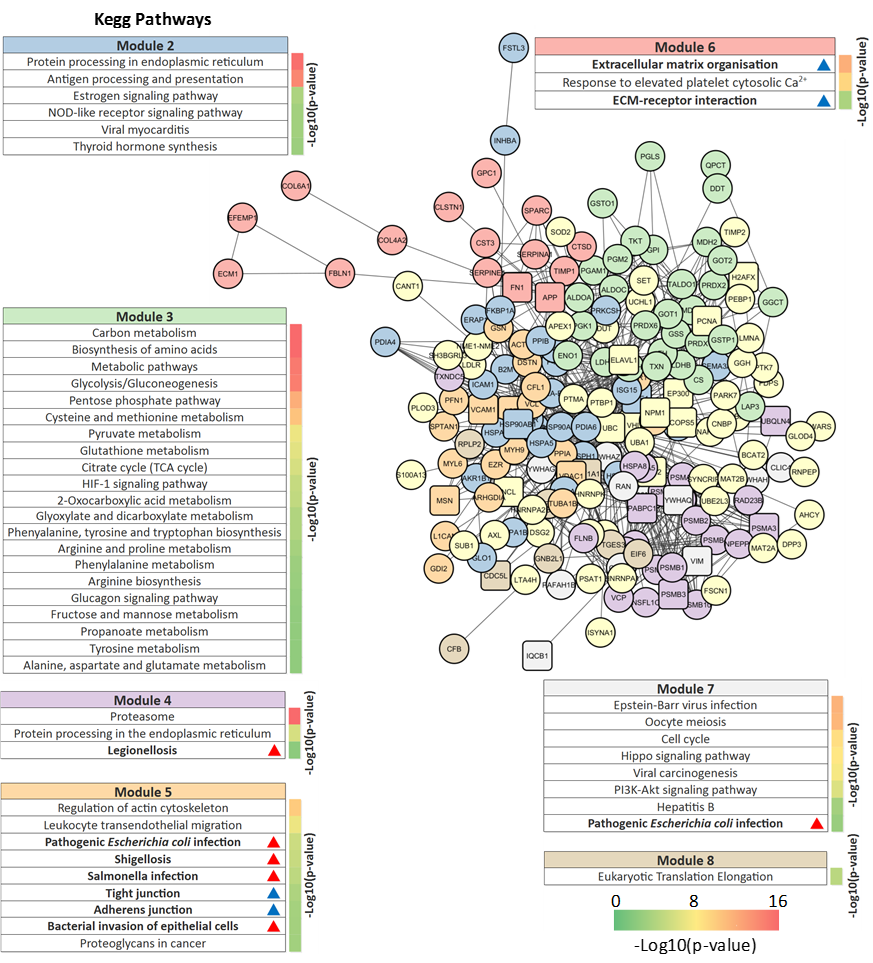


**Supplementary Figure 7. A protein–protein interaction network (PPIN) for the down-regulated proteins in *S. maltophilia* CS treated CFBE41o- cells.** The PPIN was generated using STRING v10.0 using seed proteins (circles) and candidate proteins (squares). Modules were created using the Reactome FIViz app in Cytoscape v3.4.0 and were analysed using the ‘Analyze module function’ to enrich biological pathways and assigned a unique colour. Only KEGG pathways that had an FDR < 0.01 were included in the final analysis. Annotated pathways and the weight of their associated -Log10(p-value) are highlighted next to their respective pathway as denoted by the –Log10(p-value) color key. KEGG pathways relating to bacterial infection are indicated by a red triangle and those relating to biological adhesion by a blue triangle.

Supplementary Table 1 Basic information of identified proteins.

| **Uniprot ID** | **Gene name** | **Gene ID** | **Protein name** | **Position** | **­Exp**  **↑/↓** | **SignalP^a^** | **SecP^b^** | **Phobius^c^** | **TMHMM^d^** | **WoLF Psort^e^** | |
| --- | --- | --- | --- | --- | --- | --- | --- | --- | --- | --- | --- |
|  |  |  |  |  |  | **D-Sc** | **NN-Sc** | **TM/SP** | **ExpAA** | **Loc** | **kNN** |
| A0A024RA52 | PSMA2 | 5683 | Proteasome subunit alpha type | 7p13 | ↓ | 0.13 | 0.33 | - | 1.66 | Cyto | 16.5 |
| A0A087WXM8 | BCAM | 4059 | Basal cell adhesion molecule | 19q13.2 | ↓ | 0.742 | 0.207 | TM, SP | 22.91 | Extr | 11.5 |
| A0A087X0S5 | COL6A1 | 1291 | Collagen alpha-1(VI) chain | 21q22.3 | ↓ | 0.746 | 0.234 | SP | 0 | Mito | 13 |
| A0A0A0MSA9 | PVR | 5817 | Poliovirus receptor | 19q13.2 | ↓ | 0.934 | 0.638 | TM, SP | 33.56 | Plas | 18.5 |
| A0A0C4DFU2 | SOD2 | 6648 | Superoxide dismutase | 6q25.3 | ↓ | 0.159 | 0.536 | - | 0.05 | Mito | 23 |
| A0A0G2JI36 | HLA-A | 3105 | HLA class I histocompatibility antigen, A-3 alpha chain | 6p21.3 | ↓ | 0.851 | 0.911 | SP | 7.38 | Extr | 15 |
| B1AHL2 | FBLN1 | 2192 | Fibulin-1 | 22q13.31 | ↓ | 0.867 | 0.575 | SP | 8.86 | Extr | 26.5 |
| B4DJV2 | CS | 1431 | Citrate synthase | 12q13.2 | ↓ | 0.209 | 0.602 | TM, SP | 2.16 | Mito | 21 |
| B4DY09 | ILF2 | 3608 | Interleukin enhancer-binding factor 2 | 1q21.3 | ↑ | 0.111 | 0.448 | - | 0.09 | Cyto | 13 |
| B8ZZQ6 | PTMA | 5757 | Prothymosin alpha | 2q37.1 | ↓ | 0.103 | 0.602 | - | 0 | Cyto | 18.5 |
| E7EQR4 | EZR | 7430 | Ezrin | 6q25.3 | ↓ | 0.107 | 0.567 | - | 0 | Cyto | 16.5 |
| E7ET40 | PLAU | 5328 | Urokinase-type plasminogen activator | 10q22.2 | ↑ | 0.109 | 0.454 | - | 1.26 | Cyto | 14.5 |
| E7EU96 | CSNK2A1 | 1457 | Casein kinase II subunit alpha | 20p13 | ↑ | 0.102 | 0.741 | - | 0.01 | Nucl | 25 |
| E9PKG1 | PRMT1 | 3276 | Protein arginine N-methyltransferase 1 | 19q13.3 | ↓ | 0.1 | 0.416 | - | 0.38 | Cyto | 24.5 |
| E9PLK3 | NPEPPS | 9520 | Puromycin-sensitive aminopeptidase | 17q21 | ↓ | 0.147 | 0.46 | - | 0.03 | Mito | 22.5 |
| E9PR17 | CD59 | 966 | CD59 glycoprotein | 11p13 | ↑ | 0.879 | 0.93 | SP | 8.76 | Extr | 29 |
| F6TLX2 | GLOD4 | 51031 | Glyoxalase domain-containing protein 4 | 17p13.3 | ↓ | 0.104 | 0.365 | - | 0.62 | Mito | 17 |
| F8VY35 | NAP1L1 | 4673 | Nucleosome assembly protein 1-like 1 (Fragment) | 12q21.2 | ↓ | 0.107 | 0.364 | - | 0 | Cyto | 21.5 |
| F8W1R7 | MYL6 | 4637 | Myosin light polypeptide 6 | 12q13.2 | ↓ | 0.1 | 0.423 | - | 0 | Cyto | 14 |
| F8W6I7 | HNRNPA1 | 3178 | Heterogeneous nuclear ribonucleoprotein A1 | 12q13.1 | ↓ | 0.131 | 0.085 | - | 0 | Nucl | 32 |
| G3V3X5 | LTBP2 | 4053 | Latent-transforming growth factor beta-binding protein 2 | 14q24 | ↑ | 0.65 | 0.387 | SP | 3.99 | Mito | 13 |
| G3V4W0 | HNRNPC | 3183 | Heterogeneous nuclear ribonucleoproteins C1/C2 (Fragment) | 14q11.2 | ↑ | 0.125 | 0.234 | - | 0.02 | Nucl | 31 |
| H0YNW5 | DUT | 1854 | Deoxyuridine 5-triphosphate nucleotidohydrolase, mitochondrial | 15q21.1 | ↓ | 0.116 | 0.68 | - | 0 | Cyto | 16.5 |
| J3KTF8 | ARHGDIA | 396 | Rho GDP-dissociation inhibitor 1 (Fragment) | 17q25.3 | ↓ | 0.105 | 0.867 | - | 0.01 | Cyto | 19.5 |
| M0QZP4 | BCAT2 | 587 | Branched-chain-amino-acid aminotransferase | 19q13 | ↓ | 0.125 | 0.582 | - | 1.9 | Cyto | 12.5 |
| O00299 | CLIC1 | 1192 | Chloride intracellular channel protein 1 | 6p21.3 | ↓ | 0.198 | 0.395 | - | 0.1 | Cyto | 25 |
| O00391 | QSOX1 | 5768 | Sulfhydryl oxidase 1 | 1q24 | ↓ | 0.927 | 0.611 | TM, SP | 35.29 | Golg | 13 |
| O00468 | AGRN | 375790 | Agrin | 1p36.33 | ↑ | 0.75 | 0.293 | SP | 3.24 | Extr | 28 |
| O00622 | CYR61 | 3491 | Protein CYR61 | 1p22.3 | ↑ | 0.902 | 0.772 | SP | 0.46 | Extr | 26 |
| O14818 | PSMA7 | 5688 | Proteasome subunit alpha type-7 | 20q13.33 | ↓ | 0.106 | 0.248 | - | 0.01 | Cyto | 20 |
| O15031 | PLXNB2 | 23654 | Plexin-B2 | 22q13.33 | ↑ | 0.778 | 0.588 | TM, SP | 33.83 | Plas | 12.5 |
| O43405 | COCH | 1690 | Cochlin | 14q11.2-q13 | ↑ | 0.783 | 0.655 | SP | 4.27 | Extr | 12 |
| O60506 | SYNCRIP | 10492 | Heterogeneous nuclear ribonucleoprotein Q | 6q14-q15 | ↓ | 0.102 | 0.081 | - | 0 | Nucl | 31.5 |
| O60568 | PLOD3 | 8985 | Procollagen-lysine,2-oxoglutarate 5-dioxygenase 3 | 7q22 | ↓ | 0.827 | 0.561 | SP | 0.05 | Extr | 18 |
| O75083 | WDR1 | 9948 | WD repeat-containing protein 1 | 4p16.1 | ↓ | 0.113 | 0.518 | - | 0.72 | Cyto | 22.5 |
| O75223 | GGCT | 79017 | Gamma-glutamylcyclotransferase | 7p15-p14 | ↓ | 0.102 | 0.503 | - | 0.01 | Cyto | 19 |
| O75326 | SEMA7A | 8482 | Semaphorin-7A | 15q22.3-q23 | ↓ | 0.623 | 0.484 | TM, SP | 12.32 | Nucl | 9 |
| O75367 | H2AFY | 9555 | Core histone macro-H2A.1 | 5q31.1 | ↑ | 0.116 | 0.405 | - | 6.27 | Mito | 26.5 |
| O75369 | FLNB | 2317 | Filamin-B | 3p14.3 | ↓ | 0.16 | 0.359 |  | 26.26 | Plas | 29 |
| O75882 | ATRN | 8455 | Attractin | 20p13 | ↓ | 0.105 | 0.298 | - | 0.52 | Cyto  Nucl | 17.17 |
| O76061 | STC2 | 8614 | Stanniocalcin-2 | 5q35.1 | ↓ | 0.724 | 0.601 | SP | 0 | Extr | 25 |
| O94760 | DDAH1 | 23576 | N(G),N(G)-dimethylarginine dimethylaminohydrolase 1 | 1p22 | ↓ | 0.177 | 0.47 | - | 0 | Cyto | 16 |
| O94985 | CLSTN1 | 22883 | Calsyntenin-1 | 1p36.22 | ↓ | 0.83 | 0.436 | TM, SP | 30.35 | Plas | 8 |
| O95274 | LYPD3 | 27076 | Ly6/PLAUR domain-containing protein 3 | 19q13.31 | ↑ | 0.945 | 0.635 | TM, SP | 13.24 | Extr | 30 |
| O95336 | PGLS | 25796 | 6-phosphogluconolactonase | 19p13.2 | ↓ | 0.246 | 0.87 | - | 0.05 | Extr | 14 |
| O95633 | FSTL3 | 10272 | Follistatin-related protein 3 | 19p13 | ↓ | 0.724 | 0.9 | - | 1.17 | Extr | 20 |
| O95831 | AIFM1 | 9131 | Apoptosis-inducing factor 1, mitochondrial | Xq26.1 | ↑ | 0.218 | 0.438 | TM | 4.67 | Mito | 10 |
| O95865 | DDAH2 | 23564 | N(G),N(G)-dimethylarginine dimethylaminohydrolase 2 | 6p21.3 | ↓ | 0.136 | 0.598 | - | 0 | Cyto  Nucl | 13.83 |
| P00338 | LDHA | 3939 | L-lactate dehydrogenase A chain | 11p15.4 | ↓ | 0.1 | 0.549 | - | 9.84 | Cyto | 26.5 |
| P00441 | SOD1 | 6647 | Superoxide dismutase [Cu-Zn] | 21q22.11 | ↑ | 0.123 | 0.648 | - | 0 | Cyto | 23.5 |
| P00505 | GOT2 | 2806 | Aspartate aminotransferase, mitochondrial | 16q21 | ↓ | 0.306 | 0.505 | SP | 0.2 | Mito | 27 |
| P00533 | EGFR | 1956 | Epidermal growth factor receptor | 7p12 | ↑ | 0.868 | 0.512 | TM, SP | 42.3 | Extr | 11 |
| P00558 | PGK1 | 5230 | Phosphoglycerate kinase 1 | Xq13.3 | ↓ | 0.099 | 0.389 | - | 0.03 | Cyto | 26 |
| P00751 | CFB | 629 | Complement factor B | 6p21.3 | ↓ | 0.743 | 0.457 | SP | 5.88 | Extr | 21 |
| P01009 | SERPINA1 | 5265 | Alpha-1-antitrypsin | 14q32.1 | ↓ | 0.916 | 0.852 | SP | 21.35 | Extr | 17 |
| P01024 | C3 | 718 | Complement C3 | 19p13.3-p13.2 | ↑ | 0.905 | 0.618 | SP | 8.21 | E.r. | 13 |
| P01034 | CST3 | 1471 | Cystatin-C | 20p11.21 | ↓ | 0.898 | 0.937 | SP | 17.97 | Extr | 31 |
| P01130 | LDLR | 3949 | Low-density lipoprotein receptor | 19p13.2 | ↓ | 0.849 | 0.475 | TM, SP | 21.93 | Pero | 13.5 |
| P02545 | LMNA | 4000 | Prelamin-A/C | 1q22 | ↓ | 0.123 | 0.077 | - | 0 | Nucl | 28 |
| P02751 | FN1 | 2335 | Fibronectin | 2q34 | ↑ | 0.598 | 0.369 | SP | 0.17 | Extr | 17 |
| P04075 | ALDOA | 226 | Fructose-bisphosphate aldolase A | 16p11.2 | ↓ | 0.1 | 0.356 | - | 0 | Cyto | 26 |
| P04406 | GAPDH | 2597 | Glyceraldehyde-3-phosphate dehydrogenase | 12p13 | ↓ | 0.14 | 0.467 | - | 0.05 | Cyto | 28 |
| P05023 | ATP1A1 | 476 | Sodium/potassium-transporting ATPase subunit alpha-1 | 1p21 | ↑ | 0.102 | 0.591 |  | 193.43 | Plas | 31 |
| P05121 | SERPINE1 | 5054 | Plasminogen activator inhibitor 1 | 7q22.1 | ↓ | 0.797 | 0.644 | SP | 2.53 | Extr | 25 |
| P05161 | ISG15 | 9636 | Ubiquitin-like protein ISG15 | 1p36.33 | ↓ | 0.152 | 0.702 | - | 0 | Nucl | 15 |
| P05362 | ICAM1 | 3383 | Intercellular adhesion molecule 1 | 19p13.2 | ↓ | 0.819 | 0.422 | TM, SP | 23.27 | Extr | 12 |
| P05387 | RPLP2 | 6181 | 60S acidic ribosomal protein P2 | 11p15.5 | ↓ | 0.203 | 0.265 | SP | 0.18 | Mito | 11.5 |
| P06396 | GSN | 2934 | Gelsolin | 9q33 | ↓ | 0.844 | 0.553 | SP | 5.99 | E.r. | 16 |
| P06733 | ENO1 | 2023 | Alpha-enolase | 1p36.2 | ↓ | 0.114 | 0.536 | - | 0.06 | Cyto | 26.5 |
| P06744 | GPI | 2821 | Glucose-6-phosphate isomerase | 19q13.1 | ↓ | 0.103 | 0.453 | - | 1.09 | Cyto | 22 |
| P06748 | NPM1 | 4869 | Nucleophosmin | 5q35.1 | ↑ | 0.113 | 0.811 | - | 0 | Nucl | 27 |
| P06865 | HEXA | 3073 | Beta-hexosaminidase subunit alpha | 15q24.1 | ↓ | 0.916 | 0.701 | SP | 8.81 | Mito | 15 |
| P07195 | LDHB | 3945 | L-lactate dehydrogenase B chain | 12p12.2-p12.1 | ↓ | 0.137 | 0.569 | - | 0.24 | Cyto | 17.5 |
| P07237 | P4HB | 5034 | Protein disulfide-isomerase | 17q25 | ↓ | 0.794 | 0.679 | SP | 0 | Extr | 16 |
| P07339 | CTSD | 1509 | Cathepsin D | 11p15.5 | ↓ | 0.781 | 0.758 | SP | 12.29 | Extr | 21 |
| P07355 | ANXA2 | 302 | Annexin A2 | 15q22.2 | ↑ | 0.127 | 0.746 | - | 0.01 | Cyto | 14 |
| P07384 | CAPN1 | 823 | Calpain-1 catalytic subunit | 11q13 | ↑ | 0.112 | 0.554 | - | 0.01 | Nucl | 14 |
| P07737 | PFN1 | 5216 | Profilin-1 | 17p13.3 | ↓ | 0.117 | 0.469 | - | 0.29 | Cyto | 23 |
| P07900 | HSP90AA1 | 3320 | Heat shock protein HSP 90-alpha | 14q32.33 | ↓ | 0.105 | 0.173 | - | 0.3 | Cyto | 21 |
| P07942 | LAMB1 | 3912 | Laminin subunit beta-1 | 7q22 | ↑ | 0.846 | 0.379 | SP | 0 | Extr | 29 |
| P07954 | FH | 2271 | Fumarate hydratase, mitochondrial | 1q42.1 | ↓ | 0.239 | 0.551 | SP | 2.86 | Mito | 30.5 |
| P07996 | THBS1 | 7057 | Thrombospondin-1 | 15q15 | ↑ | 0.742 | 0.345 | SP | 0.06 | Extr | 12 |
| P08476 | INHBA | 3624 | Inhibin beta A chain | 7p15-p13 | ↓ | 0.874 | 0.814 | SP | 0.01 | Mito | 12 |
| P08572 | COL4A2 | 1284 | Collagen alpha-2(IV) chain | 13q34 | ↓ | 0.74 | 0.068 | SP | 19.98 | Extr | 11 |
| P08582 | MELTF | 4241 | Melanotransferrin | 3q28-q29 | ↓ | 0.898 | 0.701 | SP | 0.16 | Extr | 24 |
| P09211 | GSTP1 | 2950 | Glutathione S-transferase P | 11q13 | ↓ | 0.228 | 0.545 | - | 0.95 | Mito | 19 |
| P09486 | SPARC | 6678 | SPARC | 5q31.3-q32 | ↓ | 0.939 | 0.942 | SP | 0.01 | Extr | 26 |
| P09525 | ANXA4 | 307 | Annexin A4 | 2p13 | ↑ | 0.103 | 0.466 | - | 0.01 | Cyto | 18 |
| P09874 | PARP1 | 142 | Poly [ADP-ribose] polymerase 1 | 1q41-q42 | ↑ | 0.102 | 0.112 | - | 0.01 | Nucl | 32 |
| P09936 | UCHL1 | 7345 | Ubiquitin carboxyl-terminal hydrolase isozyme L1 | 4p14 | ↓ | 0.133 | 0.53 | - | 0.2 | Cyto | 15 |
| P09960 | LTA4H | 4048 | Leukotriene A-4 hydrolase | 12q22 | ↓ | 0.113 | 0.485 | - | 0.07 | Cyto | 17 |
| P09972 | ALDOC | 230 | Fructose-bisphosphate aldolase C | 17cen-q12 | ↓ | 0.104 | 0.323 | - | 0 | Cyto | 17 |
| P0DMV9 | HSPA1B | 3304 | Heat shock 70 kDa protein 1B | 6p21.3 | ↓ | 0.172 | 0.28 | - | 0.28 | Cyto | 19 |
| P10599 | TXN | 7295 | Thioredoxin | 9q31 | ↓ | 0.106 | 0.37 | - | 0 | Extr | 15 |
| P11021 | HSPA5 | 3309 | 78 kDa glucose-regulated protein | 9q33.3 | ↓ | 0.898 | 0.745 | SP | 0 | E.r. | 9 |
| P11142 | HSPA8 | 3312 | Heat shock cognate 71 kDa protein | 11q24.1 | ↓ | 0.136 | 0.229 | - | 0.04 | Cyto | 15.5 |
| P11717 | IGF2R | 3482 | Cation-independent mannose-6-phosphate receptor | 6q26 | ↑ | 0.779 | 0.29 | TM, SP | 23.48 | Plas | 7 |
| P12956 | XRCC6 | 2547 | X-ray repair cross-complementing protein 6 | 22q13.2 | ↑ | 0.105 | 0.372 | - | 0.03 | Cyto | 13 |
| P13639 | EEF2 | 1938 | Elongation factor 2 | 19p13.3 | ↓ | 0.109 | 0.38 | - | 0.16 | Cyto | 21.5 |
| P13667 | PDIA4 | 9601 | Protein disulfide-isomerase A4 | 7q35 | ↓ | 0.873 | 0.543 | SP | 14.54 | Extr | 18 |
| P14314 | PRKCSH | 5589 | Glucosidase 2 subunit beta | 19p13.2 | ↓ | 0.825 | 0.308 | SP | 0 | Extr | 19 |
| P14324 | FDPS | 2224 | Farnesyl pyrophosphate synthase | 1q22 | ↓ | 0.343 | 0.639 |  | 24.84 | Mito | 27.5 |
| P14625 | HSP90B1 | 7184 | Endoplasmin | 12q24.2-q24.3 | ↓ | 0.915 | 0.495 | SP | 0.28 | E.r. | 22.5 |
| P15121 | AKR1B1 | 231 | Aldose reductase | 7q35 | ↓ | 0.184 | 0.395 | - | 0 | Cyto | 21 |
| P16035 | TIMP2 | 7077 | Metalloproteinase inhibitor 2 | 17q25 | ↓ | 0.938 | 0.854 | SP | 0.24 | Extr | 28 |
| P16070 | CD44 | 960 | CD44 antigen | 11p13 | ↑ | 0.916 | 0.233 | TM, SP | 24.71 | Extr  Plas | 9.5 |
| P17174 | GOT1 | 2805 | Aspartate aminotransferase, cytoplasmic | 10q24.1-q25.1 | ↓ | 0.14 | 0.439 | - | 0 | Cyto | 14.5 |
| P17931 | LGALS3 | 3958 | Galectin-3 | 14q22.3 | ↑ | 0.116 | 0.77 | - | 0.1 | Cyto  Nucl | 15.5 |
| P18206 | VCL | 7414 | Vinculin | 10q22.2 | ↓ | 0.126 | 0.181 | - | 0 | Cyto | 26 |
| P18669 | PGAM1 | 5223 | Phosphoglycerate mutase 1 | 10q25.3 | ↓ | 0.117 | 0.407 | - | 0 | Cyto | 20 |
| P19021 | PAM | 5066 | Peptidyl-glycine alpha-amidating monooxygenase | 5q14-q21 | ↑ | 0.838 | 0.362 | TM, SP | 28.42 | Plas | 21 |
| P19883 | FST | 10468 | Follistatin | 5q11.2 | ↑ | 0.78 | 0.493 | SP | 0.03 | Extr | 30 |
| P20618 | PSMB1 | 5689 | Proteasome subunit beta type-1 | 6q27 | ↓ | 0.112 | 0.473 | - | 4.79 | Cyto | 20 |
| P21291 | CSRP1 | 1465 | Cysteine and glycine-rich protein 1 | 1q32 | ↓ | 0.138 | 0.246 | - | 0 | Nucl | 30 |
| P21333 | FLNA | 2316 | Filamin-A | Xq28 | ↑ | 0.115 | 0.446 | TM | 1.12 | Cyto | 14.5 |
| P21589 | NT5E | 4907 | 5-nucleotidase | 6q14-q21 | ↑ | 0.873 | 0.764 | TM, SP | 34.51 | Pero | 18 |
| P22314 | UBA1 | 7317 | Ubiquitin-like modifier-activating enzyme 1 | Xp11.23 | ↓ | 0.101 | 0.53 | TM | 9.8 | Nucl | 12.5 |
| P22626 | HNRNPA2B1 | 3181 | Heterogeneous nuclear ribonucleoproteins A2/B1 | 7p15 | ↓ | 0.104 | 0.081 | - | 0.03 | Nucl | 32 |
| P23284 | PPIB | 5479 | Peptidyl-prolyl cis-trans isomerase B | 15q21-q22 | ↓ | 0.472 | 0.853 | SP | 20.43 | Extr | 25 |
| P23381 | WARS | 7453 | Tryptophan--tRNA ligase, cytoplasmic | 14q32.31 | ↓ | 0.142 | 0.4 | - | 0 | Cyto | 12 |
| P23526 | AHCY | 191 | Adenosylhomocysteinase | 20q11.22 | ↓ | 0.12 | 0.507 | - | 0.11 | Cyto | 23.5 |
| P23528 | CFL1 | 1072 | Cofilin-1 | 11q13 | ↓ | 0.102 | 0.628 | - | 0 | Mito | 17 |
| P24821 | TNC | 3371 | Tenascin | 9q33 | ↑ | 0.837 | 0.468 | SP | 15.69 | Extr | 26 |
| P25786 | PSMA1 | 5682 | Proteasome subunit alpha type-1 | 11p15.1 | ↓ | 0.104 | 0.371 | - | 0.03 | Cyto | 18 |
| P25789 | PSMA4 | 5685 | Proteasome subunit alpha type-4 | 15q25.1 | ↓ | 0.108 | 0.209 | - | 1.15 | Mito | 16 |
| P25942 | CD40 | 958 | Tumor necrosis factor receptor superfamily member 5 | 20q12-q13.2 | ↑ | 0.81 | 0.223 | TM, SP | 21.46 | Extr | 23 |
| P26599 | PTBP1 | 5725 | Polypyrimidine tract-binding protein 1 | 19p13.3 | ↓ | 0.109 | 0.415 | - | 0.11 | Cyto  Nucl | 16 |
| P26641 | EEF1G | 1937 | Elongation factor 1-gamma | 11q12.3 | ↑ | 0.126 | 0.353 | - | 1.37 | Cyto | 13.5 |
| P27695 | APEX1 | 328 | DNA-(apurinic or apyrimidinic site) lyase | 14q11.2 | ↓ | 0.105 | 0.655 | - | 0.04 | Nucl | 17.5 |
| P27797 | CALR | 811 | Calreticulin | 19p13.13 | ↓ | 0.904 | 0.366 | SP | 1.09 | E.r. | 26 |
| P27824 | CANX | 821 | Calnexin | 5q35 | ↑ | 0.863 | 0.127 | TM, SP | 22.57 | E.r. | 25 |
| P28066 | PSMA5 | 5686 | Proteasome subunit alpha type-5 | 1p13 | ↓ | 0.101 | 0.485 | - | 0 | Cyto | 19.5 |
| P28070 | PSMB4 | 5692 | Proteasome subunit beta type-4 | 1q21 | ↓ | 0.134 | 0.565 | - | 0.64 | Cyto  Nucl | 19 |
| P28074 | PSMB5 | 5693 | Proteasome subunit beta type-5 | 14q11.2 | ↓ | 0.129 | 0.726 | - | 0.09 | Nucl | 14.5 |
| P28838 | LAP3 | 51056 | Cytosol aminopeptidase | 4p15.32 | ↓ | 0.212 | 0.463 | - | 0.16 | Mito | 26 |
| P29279 | CTGF | 1490 | Connective tissue growth factor | 6q23.1 | ↑ | 0.879 | 0.722 | SP | 0.47 | Extr | 31 |
| P29317 | EPHA2 | 1969 | Ephrin type-A receptor 2 | 1p36 | ↑ | 0.833 | 0.476 | TM, SP | 32.78 | Plas | 23 |
| P29401 | TKT | 7086 | Transketolase | 3p14.3 | ↓ | 0.117 | 0.315 | - | 0.61 | Cyto | 18 |
| P30041 | PRDX6 | 9588 | Peroxiredoxin-6 | 1q25.1 | ↓ | 0.117 | 0.378 | - | 0 | Mito | 13 |
| P30046 | DDT | 1652 | D-dopachrome decarboxylase | 22q11.23 | ↓ | 0.114 | 0.479 | - | 0.01 | Cyto | 26.5 |
| P30086 | PEBP1 | 5037 | Phosphatidylethanolamine-binding protein 1 | 12q24.23 | ↓ | 0.099 | 0.672 | - | 0 | Cyto | 18.5 |
| P30101 | PDIA3 | 2923 | Protein disulfide-isomerase A3 | 15q15 | ↓ | 0.887 | 0.554 | SP | 2.52 | E.r. | 15.5 |
| P30405 | PPIF | 10105 | Peptidyl-prolyl cis-trans isomerase F, mitochondrial | 10q22.3 | ↓ | 0.296 | 0.683 | - | 0 | Mito | 23.5 |
| P30459 | HLA-A | 3105 | HLA class I histocompatibility antigen, A-74 alpha chain | 6p21.3 | ↓ | 0.894 | 0.191 | TM, SP | 25.95 | Extr | 10 |
| P30466 | HLA-B | 3106 | HLA class I histocompatibility antigen, B-18 alpha chain | 6p21.3 | ↓ | 0.869 | 0.196 | TM, SP | 28.99 | Extr | 12 |
| P30530 | AXL | 558 | Tyrosine-protein kinase receptor UFO | 19q13.1 | ↓ | 0.814 | 0.363 | TM, SP | 23.96 | Plas | 8.5 |
| P31150 | GDI1 | 2664 | Rab GDP dissociation inhibitor alpha | Xq28 | ↓ | 0.124 | 0.429 | - | 7.14 | E.r. | 9 |
| P31153 | MAT2A | 4144 | S-adenosylmethionine synthase isoform type-2 | 2p11.2 | ↓ | 0.128 | 0.552 | - | 0.41 | Cyto | 25.5 |
| P31942 | HNRNPH3 | 3189 | Heterogeneous nuclear ribonucleoprotein H3 | 10q22 | ↑ | 0.125 | 0.104 | - | 0.13 | Cyto | 16 |
| P31946 | YWHAB | 7529 | 14-3-3 protein beta/alpha | 20q13.1 | ↓ | 0.101 | 0.352 | - | 0.04 | Cyto | 29.5 |
| P32004 | L1CAM | 3897 | Neural cell adhesion molecule L1 | Xq28 | ↓ | 0.712 | 0.554 | TM, SP | 23.32 | Pero | 18 |
| P32119 | PRDX2 | 7001 | Peroxiredoxin-2 | 19p13.2 | ↓ | 0.118 | 0.522 | - | 12.94 | Cyto | 24.5 |
| P34932 | HSPA4 | 3308 | Heat shock 70 kDa protein 4 | 5q31.1 | ↓ | 0.191 | 0.226 | - | 0 | Cyto | 15.5 |
| P35052 | GPC1 | 2817 | Glypican-1 | 2q35-q37 | ↓ | 0.838 | 0.33 | TM, SP | 2.77 | Extr | 17.5 |
| P35579 | MYH9 | 4627 | Myosin-9 | 22q13.1 | ↓ | 0.105 | 0.075 | - | 2.06 | Cyto  Nucl | 15.83 |
| P35606 | COPB2 | 9276 | Coatomer subunit beta | 3q23 | ↑ | 0.104 | 0.458 | - | 0.02 | Cyto | 15.5 |
| P37837 | TALDO1 | 6888 | Transaldolase | 11p15.5-p15.4 | ↓ | 0.108 | 0.394 | - | 1.3 | Mito | 14 |
| P38646 | HSPA9 | 3313 | Stress-70 protein, mitochondrial | 5q31.1 | ↓ | 0.213 | 0.28 | - | 0.02 | Mito | 26 |
| P40306 | PSMB10 | 5699 | Proteasome subunit beta type-10 | 16q22.1 | ↓ | 0.111 | 0.515 | - | 0.06 | Cyto | 17.5 |
| P40925 | MDH1 | 4190 | Malate dehydrogenase, cytoplasmic | 2p13.3 | ↓ | 0.164 | 0.455 | - | 9.69 | Cyto | 15 |
| P40926 | MDH2 | 4191 | Malate dehydrogenase, mitochondrial | 7cen-q22 | ↓ | 0.174 | 0.644 | - | 0.08 | Mito | 30 |
| P43034 | PAFAH1B1 | 5048 | Platelet-activating factor acetylhydrolase IB subunit alpha | 17p13.3 | ↓ | 0.101 | 0.356 | - | 0.01 | Cyto  Nucl | 16 |
| P48307 | TFPI2 | 7980 | Tissue factor pathway inhibitor 2 | 7q22 | ↑ | 0.812 | 0.473 | SP | 0.25 | Extr | 28 |
| P48637 | GSS | 2937 | Glutathione synthetase | 20q11.2 | ↓ | 0.123 | 0.484 | - | 0.1 | Cyto | 15.5 |
| P49721 | PSMB2 | 5690 | Proteasome subunit beta type-2 | 1p34.2 | ↓ | 0.109 | 0.398 | - | 1.8 | Cyto | 19 |
| P50395 | GDI2 | 2665 | Rab GDP dissociation inhibitor beta | 10p15 | ↓ | 0.132 | 0.315 | - | 8.11 | E.r. | 12 |
| P52799 | EFNB2 | 1948 | Ephrin-B2 | 13q33 | ↑ | 0.358 | 0.132 | TM, SP | 32.4 | Pero | 11 |
| P53999 | SUB1 | 10923 | Activated RNA polymerase II transcriptional coactivator p15 | 5p13.3 | ↓ | 0.101 | 0.671 | - | 0 | Nucl | 23 |
| P54727 | RAD23B | 5887 | UV excision repair protein RAD23 homolog B | 9q31.2 | ↓ | 0.102 | 0.148 | - | 0 | Cyto | 18.5 |
| P55058 | PLTP | 5360 | Phospholipid transfer protein | 20q13.12 | ↓ | 0.922 | 0.7 | SP | 0.19 | Extr | 13 |
| P55072 | VCP | 7415 | Transitional endoplasmic reticulum ATPase | 9p13.3 | ↓ | 0.098 | 0.163 | - | 0 | Cyto  Nucl | 16.67 |
| P56537 | EIF6 | 3692 | Eukaryotic translation initiation factor 6 | 20q12 | ↓ | 0.121 | 0.584 | - | 0.22 | Cyto  Nucl | 12.83 |
| P56747 | CLDN6 | 9074 | Claudin-6 | 16p13.3 | ↑ | 0.596 | 0.126 | SP | 106.68 | Plas | 31 |
| P60900 | PSMA6 | 5687 | Proteasome subunit alpha type-6 | 14q13 | ↓ | 0.122 | 0.429 | - | 0 | Cyto | 18.5 |
| P60981 | DSTN | 11034 | Destrin | 20p12.1 | ↓ | 0.102 | 0.622 | - | 0.01 | Mito | 25 |
| P61769 | B2M | 567 | Beta-2-microglobulin | 15q21.1 | ↓ | 0.773 | 0.907 | SP | 2.53 | Extr | 28 |
| P61978 | HNRNPK | 3190 | Heterogeneous nuclear ribonucleoprotein K | 9q21.32-q21.33 | ↓ | 0.106 | 0.177 | - | 0 | Nucl | 20 |
| P61981 | YWHAG | 7532 | 14-3-3 protein gamma | 7q11.23 | ↓ | 0.102 | 0.29 | - | 0 | Cyto | 25 |
| P62136 | PPP1CA | 5499 | Serine/threonine-protein phosphatase PP1-alpha catalytic subunit | 11q13 | ↑ | 0.112 | 0.576 | - | 0 | Cyto | 21.5 |
| P62158 | CALM1 | 801 | Calmodulin | 14q32.11 | ↓ | 0.101 | 0.676 | - | 0 | Cyto  Nucl | 16.17 |
| P62258 | YWHAE | 7531 | 14-3-3 protein epsilon | 17p13.3 | ↓ | 0.099 | 0.33 | - | 0.07 | Cyto | 25.5 |
| P62633 | CNBP | 7555 | Cellular nucleic acid-binding protein | 3q21 | ↓ | 0.112 | 0.758 | - | 0 | Extr | 16 |
| P62805 | HIST1H4A | 8359 | Histone H4 | 6p22.2 | ↑ | 0.099 | 0.408 | - | 0 | Nucl | 32 |
| P62826 | RAN | 5901 | GTP-binding nuclear protein Ran | 12q24.3 | ↓ | 0.115 | 0.582 | - | 0.02 | Cyto | 13 |
| P62937 | PPIA | 5478 | Peptidyl-prolyl cis-trans isomerase A | 7p13 | ↓ | 0.107 | 0.339 | - | 0 | Cyto | 25.5 |
| P62942 | FKBP1A | 2280 | Peptidyl-prolyl cis-trans isomerase FKBP1A | 20p13 | ↓ | 0.102 | 0.368 | - | 0.02 | Cyto | 23.5 |
| P63104 | YWHAZ | 7534 | 14-3-3 protein zeta/delta | 8q23.1 | ↓ | 0.101 | 0.252 | - | 0.06 | Cyto | 24 |
| P63244 | RACK1 | 10399 | Receptor for activated C kinase 1 | 5q35.3 | ↓ | 0.112 | 0.465 | - | 0.01 | Cyto  Nucl | 14 |
| P63261 | ACTG1 | 71 | Actin gamma 1 | 17q25 | ↓ | 0.11 | 0.505 | - | 1.25 | Cysk | 32 |
| P68036 | UBE2L3 | 7332 | Ubiquitin-conjugating enzyme E2 L3 | 22q11.21 | ↓ | 0.104 | 0.582 | - | 0 | Cyto  Nucl | 10.5 |
| P68363 | TUBA1B | 10376 | Tubulin alpha-1B chain | 12q13.12 | ↓ | 0.133 | 0.472 | - | 1.29 | Cysk | 27 |
| P78417 | GSTO1 | 9446 | Glutathione S-transferase omega-1 | 10q25.1 | ↓ | 0.106 | 0.435 | - | 0.08 | Cyto | 23.5 |
| P80188 | LCN2 | 3934 | Neutrophil gelatinase-associated lipocalin | 9q34 | ↑ | 0.873 | 0.924 | SP | 0.08 | Extr | 14 |
| P98095 | FBLN2 | 2199 | Fibulin-2 | 3p25.1 | ↑ | 0.88 | 0.512 | SP | 5.12 | Extr | 16 |
| Q01105 | SET | 6418 | Protein SET | 9q34 | ↓ | 0.112 | 0.106 | - | 0 | Nucl | 30 |
| Q01518 | CAP1 | 10487 | Adenylyl cyclase-associated protein 1 | 1p34.2 | ↑ | 0.103 | 0.429 | - | 0.06 | Cyto | 14.5 |
| Q02790 | FKBP4 | 2288 | Peptidyl-prolyl cis-trans isomerase FKBP4 | 12p13.33 | ↑ | 0.101 | 0.287 | - | 0 | Cyto | 18.5 |
| Q03405 | PLAUR | 5329 | Urokinase plasminogen activator surface receptor | 19q13 | ↑ | 0.876 | 0.737 | SP | 0.64 | Extr | 28 |
| Q04760 | GLO1 | 2739 | Lactoylglutathione lyase | 6p21.3-p21.1 | ↓ | 0.109 | 0.397 | - | 0.14 | Cyto | 23 |
| Q04917 | YWHAH | 7533 | 14-3-3 protein eta | 22q12.3 | ↓ | 0.103 | 0.362 | - | 0 | Cyto | 27 |
| Q06830 | PRDX1 | 5052 | Peroxiredoxin-1 | 1p34.1 | ↓ | 0.113 | 0.528 | - | 10.05 | Cyto | 15 |
| Q08380 | LGALS3BP | 3959 | Galectin-3-binding protein | 17q25 | ↑ | 0.785 | 0.738 | SP | 0.02 | Extr | 24 |
| Q08945 | SSRP1 | 6749 | FACT complex subunit SSRP1 | 11q12 | ↑ | 0.099 | 0.097 | - | 0 | Nucl | 23.5 |
| Q09028 | RBBP4 | 5928 | Histone-binding protein RBBP4 | 1p35.1 | ↓ | 0.117 | 0.636 | - | 0 | Cyto  Nucl | 16.5 |
| Q12805 | EFEMP1 | 2202 | EGF-containing fibulin-like extracellular matrix protein 1 | 2p16 | ↓ | 0.848 | 0.719 | SP | 0.07 | Extr | 28 |
| Q12906 | ILF3 | 3609 | Interleukin enhancer-binding factor 3 | 19p13.2 | ↓ | 0.1 | 0.057 | - | 0.01 | Nucl | 28 |
| Q13308 | PTK7 | 5754 | Inactive tyrosine-protein kinase 7 | 6p21.1-p12.2 | ↓ | 0.81 | 0.391 | TM, SP | 39.83 | Extr | 14.5 |
| Q13753 | LAMC2 | 3918 | Laminin subunit gamma-2 | 1q25-q31 | ↑ | 0.901 | 0.34 | SP | 0.26 | Extr | 20 |
| Q13813 | SPTAN1 | 6709 | Spectrin alpha chain, non-erythrocytic 1 | 9q34.11 | ↓ | 0.098 | 0.237 | - | 0 | Nucl | 15.5 |
| Q14126 | DSG2 | 1829 | Desmoglein-2 | 18q12.1 | ↓ | 0.868 | 0.375 | TM, SP | 42.38 | Plas | 17 |
| Q14315 | FLNC | 2318 | Filamin-C | 7q32-q35 | ↑ | 0.104 | 0.45 | - | 1.67 | Cyto | 12.5 |
| Q14508 | WFDC2 | 10406 | WAP four-disulfide core domain protein 2 | 20q13.12 | ↓ | 0.882 | 0.952 | SP | 20.42 | Extr | 32 |
| Q14563 | SEMA3A | 10371 | Semaphorin-3A | 7p12.1 | ↓ | 0.747 | 0.487 | SP | 0.54 | Extr | 9 |
| Q14697 | GANAB | 23193 | Neutral alpha-glucosidase AB | 11q12.3 | ↓ | 0.564 | 0.64 | SP | 20.11 | E.r. | 15 |
| Q15019 | 37500.00 | 4735 | Septin-2 | 2q37 | ↑ | 0.105 | 0.129 | - | 0.11 | Cyto | 20 |
| Q15084 | PDIA6 | 10130 | Protein disulfide-isomerase A6 | 2p25.1 | ↓ | 0.813 | 0.711 | SP | 5.76 | Extr | 17 |
| Q15185 | PTGES3 | 10728 | Prostaglandin E synthase 3 | 12q13.3\|12 | ↓ | 0.126 | 0.729 | - | 0 | Nucl | 13.5 |
| Q15582 | TGFBI | 7045 | Transforming growth factor-beta-induced protein ig-h3 | 5q31 | ↑ | 0.886 | 0.454 | SP | 15.09 | Extr | 9 |
| Q15691 | MAPRE1 | 22919 | Microtubule-associated protein RP/EB family member 1 | 20q11.1-q11.23 | ↑ | 0.106 | 0.356 | - | 0.04 | Cyto | 19.5 |
| Q15828 | CST6 | 1474 | Cystatin-M | 11q13 | ↓ | 0.845 | 0.944 | SP | 16.01 | Extr | 29 |
| Q16270 | IGFBP7 | 3490 | Insulin-like growth factor-binding protein 7 | 4q12 | ↓ | 0.828 | 0.536 | SP | 3.86 | Extr | 32 |
| Q16610 | ECM1 | 1893 | Extracellular matrix protein 1 | 1q21 | ↓ | 0.734 | 0.711 | SP | 14.44 | Extr | 25 |
| Q16651 | PRSS8 | 5652 | Prostasin | 16p11.2 | ↑ | 0.683 | 0.813 | TM, SP | 14.91 | Extr | 30 |
| Q16658 | FSCN1 | 6624 | Fascin | 7p22 | ↓ | 0.114 | 0.385 | - | 0.01 | Cyto | 15 |
| Q16769 | QPCT | 25797 | Glutaminyl-peptide cyclotransferase | 2p22.2 | ↓ | 0.832 | 0.788 | SP | 4.36 | Extr | 21 |
| Q16778 | HIST2H2BE | 8349 | Histone H2B type 2-E | 1q21.2 | ↑ | 0.104 | 0.306 | - | 0 | Nucl | 32 |
| Q16787 | LAMA3 | 3909 | Laminin subunit alpha-3 | 18q11.2 | ↑ | 0.63 | 0.328 | - | 0.92 | Extr | 27 |
| Q24JP5 | TMEM132A | 54972 | Transmembrane protein 132A | 11q12.2 | ↑ | 0.664 | 0.164 | TM, SP | 23.42 | E.r. | 9.5 |
| Q32Q12 | NME1-NME2 | 654364 | Nucleoside diphosphate kinase | 17q21.3 | ↓ | 0.371 | 0.513 | SP | 0.08 | Cyto | 21.5 |
| Q5H9A7 | TIMP1 | 7076 | Metalloproteinase inhibitor 1 | Xp11.3-p11.23 | ↓ | 0.12 | 0.412 | - | 0.46 | Extr | 23 |
| Q5T123 | SH3BGRL3 | 83442 | SH3 domain-binding glutamic acid-rich-like protein 3 | 1p36.11 | ↓ | 0.098 | 0.106 | - | 0 | Mito | 15 |
| Q5TEC6 | HIST2H3PS2 | 440686 | Histone H3 | 1q21.2 | ↑ | 0.103 | 0.742 | - | 0 | Nucl | 32 |
| Q5VT79 | ANXA8L1 | 728113 | Annexin A8-like protein 1 | 10q11.22 | ↑ | 0.123 | 0.291 | - | 0 | Cyto | 26 |
| Q5VTE0 | EEF1A1P5 | 158078 | Putative elongation factor 1-alpha-like 3 | 9q34.13 | ↓ | 0.116 | 0.154 | - | 0.07 | Cyto | 26 |
| Q6EMK4 | VASN | 114990 | Vasorin | 16p13.3 | ↓ | 0.943 | 0.216 | TM, SP | 23.42 | Extr | 9.5 |
| Q6UVK1 | CSPG4 | 1464 | Chondroitin sulfate proteoglycan 4 | 15q24.2 | ↑ | 0.835 | 0.584 | TM, SP | 24.29 | Extr | 13.5 |
| Q6UW32 | IGFL1 | 374918 | Insulin growth factor-like family member 1 | 19q13.32 | ↑ | 0.724 | 0.885 | SP | 19.67 | Extr | 32 |
| Q6YHK3 | CD109 | 135228 | CD109 antigen | 6q13 | ↑ | 0.794 | 0.6 | SP | 6.2 | E.r. | 16 |
| Q86SQ4 | ADGRG6 | 57211 | Adhesion G protein-coupled receptor G6 | 6q24.1 | ↑ | 0.807 | 0.404 | SP | 173.37 | Plas | 30 |
| Q86UY0 | TXNDC5 | 81567 | Protein BLOC1S5-TXNDC5 | 6p24.3 | ↓ | 0.103 | 0.465 | - | 0 | Cyto | 23.5 |
| Q8N474 | SFRP1 | 6422 | Secreted frizzled-related protein 1 | 8p11.21 | ↑ | 0.747 | 0.692 | SP | 19.35 | Plas | 17 |
| Q8NCW5 | NAXE | 128240 | NAD(P)H-hydrate epimerase | 1q21 | ↓ | 0.587 | 0.819 | SP | 0.01 | Mito | 20.5 |
| Q8WVQ1 | CANT1 | 124583 | Soluble calcium-activated nucleotidase 1 | 17q25.3 | ↓ | 0.149 | 0.635 | TM | 16.54 | Extr | 9 |
| Q92520 | FAM3C | 10447 | Protein FAM3C | 7q31 | ↑ | 0.309 | 0.821 | SP | 21.82 | Extr | 31 |
| Q92598 | HSPH1 | 10808 | Heat shock protein 105 kDa | 13q12.3 | ↓ | 0.129 | 0.327 | - | 0 | Cyto | 12.5 |
| Q92626 | PXDN | 7837 | Peroxidasin homolog | 2p25 | ↑ | 0.838 | 0.385 | SP | 19.22 | Extr | 13 |
| Q92820 | GGH | 8836 | Gamma-glutamyl hydrolase | 8q12.3 | ↓ | 0.827 | 0.628 | SP | 3.19 | Extr | 20 |
| Q92876 | KLK6 | 5653 | Kallikrein-6 | 19q13.3 | ↓ | 0.828 | 0.771 | SP | 3.65 | Extr | 29 |
| Q96G03 | PGM2 | 55276 | Phosphoglucomutase-2 | 4p14 | ↓ | 0.103 | 0.414 | - | 0.03 | Nucl | 15.5 |
| Q96PD2 | DCBLD2 | 131566 | Discoidin, CUB and LCCL domain-containing protein 2 | 3q12.1\|3 | ↑ | 0.445 | 0.347 | SP | 35.79 | Plas | 19 |
| Q99497 | PARK7 | 11315 | Protein deglycase DJ-1 | 1p36.23 | ↓ | 0.147 | 0.493 | - | 0.38 | Cyto | 24 |
| Q99584 | S100A13 | 6284 | Protein S100-A13 | 1q21 | ↓ | 0.1 | 0.369 | - | 0 | Cyto | 20 |
| Q99878 | HIST1H2AJ | 8331 | Histone H2A type 1-J | 6p22.1 | ↑ | 0.119 | 0.563 | - | 1.67 | Nucl | 32 |
| Q9BTY2 | FUCA2 | 2519 | Plasma alpha-L-fucosidase | 6q24 | ↓ | 0.88 | 0.779 | SP | 9.47 | Extr | 13 |
| Q9GZM7 | TINAGL1 | 64129 | Tubulointerstitial nephritis antigen-like | 1p35.2 | ↓ | 0.792 | 0.935 | SP | 1.62 | Extr | 19 |
| Q9GZN4 | PRSS22 | 64063 | Brain-specific serine protease 4 | 16p13.3 | ↓ | 0.67 | 0.744 | SP | 11.14 | Extr | 18.5 |
| Q9H4A4 | RNPEP | 6051 | Aminopeptidase B | 1q32 | ↓ | 0.129 | 0.554 | - | 0.05 | Nucl | 31.5 |
| Q9NPH2 | ISYNA1 | 51477 | Inositol-3-phosphate synthase 1 | 19p13.11 | ↓ | 0.104 | 0.55 | - | 1.12 | Nucl | 9.5 |
| Q9NY33 | DPP3 | 10072 | Dipeptidyl peptidase 3 | 11q13.2 | ↓ | 0.107 | 0.408 | - | 1.29 | Cyto | 21 |
| Q9NYU2 | UGGT1 | 56886 | UDP-glucoseglycoprotein glucosyltransferase 1 | 2q14.3 | ↑ | 0.382 | 0.632 | TM | 21.98 | Plas | 23 |
| Q9NZ08 | ERAP1 | 51752 | Endoplasmic reticulum aminopeptidase 1 | 5q15 | ↓ | 0.818 | 0.609 | SP | 3.05 | Extr | 14 |
| Q9NZL9 | MAT2B | 27430 | Methionine adenosyltransferase 2 subunit beta | 5q34 | ↓ | 0.112 | 0.519 | - | 0.01 | Cyto | 11 |
| Q9NZV1 | CRIM1 | 51232 | Cysteine-rich motor neuron 1 protein | 2p21 | ↑ | 0.826 | 0.521 | TM, SP | 32.05 | Extr | 24 |
| Q9UBP4 | DKK3 | 27122 | Dickkopf-related protein 3 | 11p15.2 | ↓ | 0.722 | 0.638 | SP | 3.04 | Extr | 31 |
| Q9UBR2 | CTSZ | 1522 | Cathepsin Z | 20q13.32 | ↓ | 0.845 | 0.861 | SP | 17.79 | Extr | 26 |
| Q9UNZ2 | NSFL1C | 55968 | NSFL1 cofactor p47 | 20p13 | ↓ | 0.111 | 0.359 | - | 0.05 | Nucl | 27 |
| Q9Y4K0 | LOXL2 | 4017 | Lysyl oxidase homolog 2 | 8p21.3 | ↓ | 0.892 | 0.711 | SP | 3.82 | Extr | 29 |
| Q9Y5B9 | SUPT16H | 11198 | FACT complex subunit SPT16 | 14q11.2 | ↑ | 0.103 | 0.126 | - | 0.06 | Nucl | 23.5 |
| Q9Y617 | PSAT1 | 29968 | Phosphoserine aminotransferase | 9q21.2 | ↓ | 0.122 | 0.384 | - | 0.12 | Cyto | 21 |
| Q9Y696 | CLIC4 | 25932 | Chloride intracellular channel protein 4 | 1p36.11 | ↓ | 0.127 | 0.612 | - | 0.14 | Cyto | 10.5 |
| X6RJP6 | TAGLN2 | 8407 | Transgelin-2 (Fragment) | 1q21-q25 | ↑ | 0.104 | 0.673 | - | 0 | Cyto  Nucl | 14.5 |

^a^Secretion prediction according to signal peptide probability of Signal P 4.0 server. D-scores (D-Sc) > 0.45 discriminate signal peptides from non-signal peptides

^b^Secretion prediction according to Secretome P 2.0 server. Proteins with NN-score (NN-Sc) ≥0.6 are predicted as secreted by non-classical secretory pathways

^c^ Transmembrane topology and signal peptide predictor according to Phobius. Abbreviations: TM, transmembrane; SP, signal peptide.

^d^ Prediction of transmembrane helices in proteins according to TMHMM server v2.0. Proteins with Exp-AA score (expected number of amino acid residues in transmembrane helices) ≥18 are predicted as transmembrane.

^e^Subcellular location prediction according to WoLF Psort. Abbreviation: Cyto, cytoskeleton; Extr, extracellular; Mito, mitochondrion; Plas, plasma membrane; ER, endoplasmic reticulum; Cysk, cytoskeleton; Golg, Golgi apparatus; Nucl, nucleus; Pero, peroxisome. kNN (k-nearest neighbour classifier) is used for prediction.

**Supplementary Table 2 Summary of interaction data from GeneMANIA**

|  | **Up-regulated (%)** | **Down-regulated (%)** |
| --- | --- | --- |
| **Co-expression** | **52.89** | **52.96** |
| **Physical interactions** | **20.62** | **30.56** |
| **Predicted** | **14.22** | **9.37** |
| **Co-localization** | **7.22** | **4.60** |
| **Shared protein domains** | **4.16** | **1.39** |
| **Pathway** | **0.75** | **0.73** |
| **Genetic interaction** | **0.12** | **0.38** |

Supplementary Table 3 Top 100 highly ranked degree-based proteins in NetworkAnalyst

|  | **Up-regulated Network** | | | | **Down-regulated Network** | | | |
| --- | --- | --- | --- | --- | --- | --- | --- | --- |
| **Rank** | **Gene** | **Uniprot ID** | **Degree** | **Betweeness** | **Gene** | **Uniprot ID** | **Degree** | **Betweeness** |
|  | UBC | F5GXK7 | 56 | 4097.6 | UBC | F5GXK7 | 169 | 22473.89 |
|  | SUMO2 | J3KRH1 | 20 | 269.67 | KIAA0101 | H0YKX3 | 66 | 1812.86 |
|  | HIST1H4E | P62805 | 19 | 390.18 | FN1 | H0Y4K8 | 63 | 1639.64 |
|  | ELAVL1 | M0QZR9 | 14 | 184.69 | SUMO2 | J3KRH1 | 58 | 1274.58 |
|  | SUMO1 | B8ZZ67 | 14 | 92.56 | APP | A0A0A0MRG2 | 47 | 1738.83 |
|  | YWHAZ | B0AZS6 | 13 | 103.41 | COPS5 | E5RFS1 | 42 | 890.68 |
|  | MYC | A0A087WUS5 | 12 | 135.21 | CALM3 | Q96HY3 | 41 | 1261.78 |
|  | HNF4A | A0A087WXV4 | 12 | 132.56 | TRAF6 | Q9Y4K3 | 41 | 588.99 |
|  | GRB2 | J3KT38 | 12 | 80.11 | IKBKE | A0A075B7B4 | 39 | 558.49 |
|  | VHL | P40337 | 12 | 74.81 | IQCB1 | C9J6Z7 | 36 | 433.6 |
|  | TRAF6 | Q9Y4K3 | 10 | 75.87 | ELAVL1 | M0QZR9 | 34 | 714.53 |
|  | XRCC5 | C9JZ81 | 10 | 39.18 | PSMA3 | G3V3W4 | 34 | 326.52 |
|  | ATF2 | B8ZZU6 | 9 | 63.81 | HNF4A | A0A087WXV4 | 33 | 672.05 |
|  | MCC | A0A096LNU0 | 9 | 40.49 | VHL | P40337 | 33 | 527.2 |
|  | IKBKE | A0A075B7B4 | 9 | 38.73 | H2AFX | P16104 | 33 | 384.79 |
|  | FBXO6 | J3KQ72 | 8 | 205.52 | YWHAQ | E9PG15 | 32 | 344.1 |
|  | EGR1 | P18146 | 8 | 72.59 | GRB2 | J3KT38 | 24 | 333.9 |
|  | DDX17 | A0A0U1RQJ0 | 7 | 62.18 | MCC | A0A096LNU0 | 24 | 198.36 |
|  | SIRT1 | B0QZ35 | 7 | 58.07 | EIF1B | O60739 | 23 | 187.69 |
|  | EEF1A1 | A0A087WV01 | 7 | 35.57 | UBD | A0A0G2JH67 | 22 | 595.73 |
|  | HLA-B | A0A140T951 | 7 | 23.52 | PAK2 | H7C1X3 | 21 | 149.68 |
|  | FMNL1 | A0A0A0MR62 | 7 | 21.74 | RAD23A | K7ELU6 | 21 | 109.94 |
|  | RELA | A0A087WVP0 | 6 | 82.32 | SP1 | H3BUU5 | 20 | 412.48 |
|  | HSPA4 | A0A087WTS8 | 6 | 36.68 | NME2 | F6XY72 | 19 | 312.99 |
|  | PIK3R1 | E5RGI8 | 6 | 23.72 | PCNA | P12004 | 17 | 126.91 |
|  | MCM3 | J3KQ69 | 6 | 11.2 | HNF1A | A0A087WYP0 | 16 | 169.82 |
|  | ABL1 | P00519 | 5 | 117.89 | FTSJ1 | B7Z4K4 | 16 | 81.74 |
|  | UCHL5 | H0Y4E0 | 5 | 64.63 | IKBKG | A0A087WUW6 | 16 | 81.37 |
|  | SMURF2 | J3QLG1 | 5 | 52.67 | CBL | A0A0U1RQX8 | 16 | 42.39 |
|  | NFKB1 | D6RC45 | 5 | 40.94 | ABL1 | P00519 | 15 | 153.96 |
|  | CFTR | C9J6L5 | 5 | 35.45 | EPB41 | P11171 | 15 | 95.91 |
|  | SHC1 | H0Y539 | 5 | 25.76 | MSN | P26038 | 15 | 77.18 |
|  | NFKB2 | A0A087WWG7 | 5 | 25.11 | PRKAB1 | F5H2X8 | 15 | 61.84 |
|  | PLCG1 | A0A0D9SEK2 | 5 | 24.81 | TNIK | C9J338 | 14 | 71.3 |
|  | NDRG1 | E5RG99 | 5 | 19.13 | MAGOH | P61326 | 14 | 66.51 |
|  | SRC | P12931 | 5 | 9.43 | PABPC1 | A0A087WTT1 | 14 | 59.1 |
|  | CSNK2B | A0A0G2JM12 | 5 | 6.66 | PSMB3 | A0A087WUL2 | 14 | 32.59 |
|  | COL1A1 | I3L3H7 | 4 | 148.71 | VIM | B0YJC4 | 13 | 63.47 |
|  | TERF2IP | H3BMI8 | 4 | 59.69 | PINX1 | E5RGR1 | 13 | 56.92 |
|  | SKIL | C9J8R9 | 4 | 58.45 | ARF6 | P62330 | 13 | 52.36 |
|  | FYN | E5RFM0 | 4 | 53.66 | MAP3K1 | Q13233 | 13 | 48.53 |
|  | MMP2 | H3BR66 | 4 | 33.79 | RIPK2 | E5RGK6 | 13 | 33.05 |
|  | APC | A0A087WYF3 | 4 | 30.44 | PSME3 | A0A087WTV2 | 13 | 33.04 |
|  | BCL2 | P10415 | 4 | 22.69 | POMP | Q9Y244 | 13 | 15.06 |
|  | LRP1 | H0YJ88 | 4 | 20.53 | TNFRSF1A | F5GWJ4 | 12 | 51.53 |
|  | TAB2 | Q9NYJ8 | 4 | 18.93 | GH1 | B1A4G9 | 12 | 44.1 |
|  | CAV1 | C9JKI3 | 4 | 18.53 | HNRNPA3 | H7C1J8 | 12 | 35.19 |
|  | MAP3K3 | J3KRN4 | 4 | 15.79 | XRCC6 | B1AHC9 | 11 | 185.48 |
|  | TRAF2 | B1AMX7 | 4 | 15.46 | EGR1 | P18146 | 11 | 80.54 |
|  | NCOR1 | A0A088AWL3 | 4 | 14.36 | DHX9 | Q08211 | 11 | 47.05 |
|  | CALM3 | M0QZ52 | 4 | 13.97 | MAP3K5 | Q99683 | 11 | 33.86 |
|  | ANXA1 | P04083 | 4 | 13.4 | TRAF2 | B1AMX7 | 11 | 25.14 |
|  | CTNNB1 | B4DGU4 | 4 | 11.24 | KIF5B | P33176 | 11 | 17.58 |
|  | TNFRSF1B | B5A977 | 4 | 10.99 | TAB2 | Q9NYJ8 | 11 | 17.5 |
|  | ANXA8L2 | Q5VT79 | 4 | 10.23 | SNCA | D6RA31 | 10 | 100.38 |
|  | TJP1 | A0A087X0K9 | 3 | 160.22 | EP300 | A0A0U1RQG3 | 10 | 82.04 |
|  | TXN | P10599 | 3 | 60.87 | SRPK1 | D6RB98 | 10 | 61.25 |
|  | TERF1 | E5RFJ5 | 3 | 25.63 | RPS3A | D6R9B6 | 10 | 35.59 |
|  | STAT1 | D2KFR9 | 3 | 24.18 | SGK1 | E9PJN2 | 10 | 31.28 |
|  | FOS | G3V289 | 3 | 18.83 | GABARAPL2 | H3BQ50 | 10 | 23.34 |
|  | ITGAM | H3BMV4 | 3 | 16.36 | ATXN1 | P54253 | 9 | 265.67 |
|  | LRP2 | E9PC35 | 3 | 15.06 | MPG | A2IDA3 | 9 | 206.85 |
|  | CEBPB | P17676 | 3 | 14.42 | CDKN1B | E7ES52 | 9 | 34.93 |
|  | SERPINA1 | A0A024R6I7 | 3 | 12.48 | TFE3 | P19532 | 9 | 28.83 |
|  | JUN | P05412 | 3 | 8.34 | TARDBP | A0A087WTG4 | 9 | 25.14 |
|  | MYCBP | A0A0D9SEI7 | 3 | 8.26 | NDRG1 | E5RG99 | 9 | 24.06 |
|  | VTN | F5GX75 | 3 | 8.04 | REL | Q04864 | 9 | 21.25 |
|  | CBL | A0A0U1RQX8 | 3 | 7.25 | UBE3A | A0A0D9SEJ2 | 9 | 16.89 |
|  | EFNA1 | P20827 | 3 | 3.75 | ECHS1 | P30084 | 8 | 41.93 |
|  | ATF7IP | A8MV73 | 2 | 159 | CRK | I3L297 | 8 | 35.31 |
|  | TK1 | K7ENW5 | 2 | 83.93 | SRSF3 | A0A087X2D0 | 8 | 30.29 |
|  | SDC2 | E5RHU3 | 2 | 22.4 | PIK3R1 | E5RGI8 | 8 | 30.23 |
|  | ACD | A0A0C4DGT6 | 2 | 19.96 | DFFA | K7ERT1 | 8 | 24.02 |
|  | BMPR2 | Q13873 | 2 | 17.65 | SFPQ | H0Y9K7 | 8 | 22.08 |
|  | TRAF4 | A0A0C4DFM9 | 2 | 14.89 | VDAC1 | C9JI87 | 8 | 21.39 |
|  | RUNX1 | A0A0C4DG58 | 2 | 11.46 | CTPS1 | P17812 | 8 | 18.86 |
|  | COL2A1 | P02458 | 2 | 10.78 | NPM1 | E5RGW4 | 8 | 18.65 |
|  | JUNB | P17275 | 2 | 8.38 | PCBP1 | Q15365 | 8 | 16.67 |
|  | POU2F1 | A0A0A0MSV5 | 2 | 8.23 | NCL | C9J1H7 | 8 | 12.28 |
|  | ITGB2 | A0A087WX36 | 2 | 5.41 | UBQLN4 | Q9NRR5 | 7 | 406.79 |
|  | ANXA7 | B9ZVT2 | 2 | 4.61 | CASP8 | A0A0A0MS31 | 7 | 69.41 |
|  | JUND | P17535 | 2 | 3.71 | TK1 | K7ENW5 | 7 | 59.93 |
|  | TFAP2A | C1K3N0 | 2 | 3.24 | CSNK2B | A0A0G2JM12 | 7 | 39.79 |
|  | LAMA5 | A0A087WYH7 | 2 | 3.01 | TANK | E7EM55 | 7 | 39.13 |
|  | TNFRSF14 | A0A0D9SF52 | 2 | 2.53 | TUFM | H3BNU3 | 7 | 23.27 |
|  | SETDB1 | B0QZE6 | 2 | 2.26 | CSE1L | P55060 | 7 | 22.88 |
|  | GFI1B | Q5VTD9 | 2 | 2.09 | UBB | B4DV12 | 7 | 14.12 |
|  | APOB | A0A087WTM7 | 2 | 2.07 | KAT5 | E9PJI1 | 7 | 13.8 |
|  | ATN1 | P54259 | 2 | 1.83 | ACTA1 | A6NL76 | 7 | 11.66 |
|  | PRKAB1 | F5H2X8 | 2 | 1.53 | CDC5L | Q99459 | 7 | 7.43 |
|  |  |  |  |  | HNRNPR | B4DT28 | 7 | 5.05 |
|  |  |  |  |  | GSK3B | P49841 | 6 | 233.22 |
|  |  |  |  |  | UBQLN1 | H0YDS0 | 6 | 103.33 |
|  |  |  |  |  | DNMT1 | K7EIZ6 | 6 | 75.48 |
|  |  |  |  |  | ANXA7 | B9ZVT2 | 6 | 39.75 |
|  |  |  |  |  | ARAF | P10398 | 6 | 27.02 |
|  |  |  |  |  | NUDC | A0A0A0MSS4 | 6 | 21.52 |
|  |  |  |  |  | SRSF1 | J3KSR8 | 6 | 18.16 |
|  |  |  |  |  | MTOR | B1AKP8 | 6 | 17.46 |
|  |  |  |  |  | RPL31 | B7Z4C8 | 6 | 11.21 |

Supplementary Table 4 Top 100 highly ranked degree-based proteins in GeneMANIA

|  | **Up-regulated Network** | | | **Down-regulated Network** | | |
| --- | --- | --- | --- | --- | --- | --- |
| **Rank** | **Gene** | **Uniprot ID** | **Score** | **Gene** | **Uniprot ID** | **Score** |
|  | UGGT2 | A6NP03 | 1.7 | VCAM1 | E9PDD2 | 4.42 |
|  | XRCC5 | C9JZ81 | 1.51 | PSMA3 | G3V3W4 | 4.08 |
|  | VCAM1 | E9PDD2 | 1.44 | PSMB3 | A0A087WUL2 | 3.96 |
|  | EEF1B2 | C9JZW3 | 1.44 | YWHAQ | E9PG15 | 3.84 |
|  | ANXA1 | P04083 | 1.38 | PSMB6 | A0A087X2I4 | 3.75 |
|  | SOD3 | M0R1V4 | 1.36 | ACTB | C9JTX5 | 3.5 |
|  | POSTN | B1ALD9 | 1.31 | PSMB7 | Q5TBG5 | 3.39 |
|  | ANXA5 | D6RBE9 | 1.29 | HSP90AB1 | P08238 | 3.34 |
|  | THBS2 | P35442 | 1.23 | PKM | B4DNK4 | 3.28 |
|  | SEPT6 | B1AMS2 | 1.22 | HSPD1 | C9J0S9 | 3.1 |
|  | ITGA3 | D6R9X8 | 1.21 | TPI1 | P60174 | 3.09 |
|  | S100A10 | P60903 | 1.21 | YBX1 | A0A087X1S2 | 3.07 |
|  | S100A11 | P31949 | 1.2 | PXN | F5GZ78 | 3.03 |
|  | EEF1D | A0A087X1X7 | 1.2 | VIM | B0YJC4 | 2.97 |
|  | ITGAD | Q13349 | 1.18 | RPLP0 | F8VPE8 | 2.94 |
|  | COL6A3 | C9JNG9 | 1.14 | ATP5B | F8W079 | 2.89 |
|  | CD248 | Q9HCU0 | 1.13 | LGALS1 | F8WCQ5 | 2.85 |
|  | LGALS1 | F8WCQ5 | 1.13 | MIF | P14174 | 2.8 |
|  | LAMB3 | Q13751 | 1.13 | CAPNS1 | A0A075B7C0 | 2.79 |
|  | LAMC1 | R4GNC7 | 1.12 | RPN1 | B7Z4L4 | 2.75 |
|  | CAP2 | A0A087WZ15 | 1.12 | MANF | A8K878 | 2.73 |
|  | FBN1 | F6U495 | 1.1 | RPN2 | F2Z3K5 | 2.7 |
|  | ANXA3 | D6RA82 | 1.1 | RHOA | C9JNR4 | 2.69 |
|  | CAPN2 | C9JWY7 | 1.07 | SLC25A5 | P05141 | 2.66 |
|  | LPHN2 | B1ALU3 | 1.07 | XRCC5 | C9JZ81 | 2.63 |
|  | ANXA7 | B9ZVT2 | 1.06 | PSMD14 | C9JW37 | 2.63 |
|  | LAMA5 | A0A087WYH7 | 1.02 | PSMC1 | G3V4X1 | 2.63 |
|  | ANXA11 | H0Y6E1 | 1.01 | EEF1A1 | A0A087WV01 | 2.63 |
|  | IER3 | P46695 | 1.01 | NME1 | C9K028 | 2.61 |
|  | CAV2 | E9PCT3 | 1 | ATP5G3 | P48201 | 2.61 |
|  | HIST1H2BD | P58876 | 0.99 | CCT2 | F5GWF6 | 2.6 |
|  | HIST1H2AC | Q93077 | 0.99 | ATP5A1 | A0A0A0MTS3 | 2.59 |
|  | COL4A1 | A0A087WTY5 | 0.98 | CSNK2B | A0A0G2JM12 | 2.54 |
|  | GLI2 | A0A0A0MR29 | 0.98 | ANXA5 | D6RBE9 | 2.49 |
|  | ITGB5 | F8WBG2 | 0.98 | CCT3 | B4DUR8 | 2.48 |
|  | ITGB1 | C9JJP8 | 0.97 | EEF1B2 | C9JZW3 | 2.46 |
|  | SERPINE1 | P05121 | 0.95 | RPLP1 | H0YL57 | 2.45 |
|  | HIST1H2BK | O60814 | 0.95 | RPL6 | F8VR69 | 2.44 |
|  | MMP2 | H3BR66 | 0.95 | HYOU1 | A0A087WW13 | 2.43 |
|  | CFI | A0A087X0I2 | 0.95 | TMSB10 | P63313 | 2.42 |
|  | VCAN | D6RGZ6 | 0.93 | PSMD8 | K7EJC1 | 2.41 |
|  | HGF | C9JDP4 | 0.93 | HSPE1 | B8ZZ54 | 2.41 |
|  | A2M | F5H1E8 | 0.92 | PHB2 | F5GWA7 | 2.4 |
|  | FLNB | A0A0A0MT44 | 0.92 | CCT7 | A0A0D9SG95 | 2.39 |
|  | AK2 | F8VPP1 | 0.92 | ACLY | K7EIE7 | 2.37 |
|  | LAMA4 | A0A0A0MQS9 | 0.92 | SNRPF | A0A0B4J254 | 2.37 |
|  | DHFR | B4DM58 | 0.91 | SLC25A3 | F8VVM2 | 2.37 |
|  | VIM | B0YJC4 | 0.91 | RPS5 | M0QZN2 | 2.35 |
|  | COL3A1 | H7C435 | 0.9 | PSMD2 | C9JPC0 | 2.34 |
|  | ELN | B3KRT8 | 0.89 | S100A11 | P31949 | 2.34 |
|  | ANXA8L2 | Q5VT79 | 0.89 | VDAC2 | A0A0A0MR02 | 2.33 |
|  | TAGLN | E9PJ32 | 0.88 | PSMC3 | E9PKD5 | 2.32 |
|  | TIMP1 | H0Y789 | 0.87 | STIP1 | F5GXD8 | 2.31 |
|  | CNN3 | E9PDU6 | 0.86 | CCT5 | B7ZAR1 | 2.31 |
|  | ITGA6 | C9JXX7 | 0.85 | SNRPD2 | K7EJB5 | 2.31 |
|  | HIST1H1C | P16403 | 0.84 | RBMX | H0Y6E7 | 2.3 |
|  | COL5A2 | A0A087WYX9 | 0.84 | PSMD1 | A0A087WW66 | 2.29 |
|  | ILF3 | K7EJ09 | 0.84 | CD63 | F8VNT9 | 2.29 |
|  | HSPG2 | A0A0U1RQT3 | 0.84 | NCL | C9J1H7 | 2.29 |
|  | HIST1H3H | P68431 | 0.84 | ATP5C1 | P36542 | 2.28 |
|  | CFH | A0A0D9SG88 | 0.83 | KARS | H3BMR9 | 2.27 |
|  | SPARC | E5RJA5 | 0.83 | COL4A1 | A0A087WTY5 | 2.26 |
|  | FILIP1L | C9JYJ6 | 0.83 | NONO | C9IZL7 | 2.26 |
|  | ITGA5 | B4E3F4 | 0.82 | CDK4 | F8VTV8 | 2.26 |
|  | LUM | P51884 | 0.82 | CSTB | P04080 | 2.26 |
|  | IGFBP6 | F8VVA5 | 0.82 | EIF4A1 | J3KRC2 | 2.25 |
|  | COL1A1 | I3L3H7 | 0.82 | UBC | F5GXK7 | 2.25 |
|  | SERPINH1 | E9PIG2 | 0.82 | VDAC1 | C9JI87 | 2.24 |
|  | MFAP2 | P55001 | 0.82 | MSN | P26038 | 2.23 |
|  | PLAT | B4DN26 | 0.81 | SNRPB | A8MT02 | 2.23 |
|  | COL1A2 | A0A087WTA8 | 0.81 | PGD | K7EJT3 | 2.23 |
|  | COL4A2 | A0A087WY39 | 0.81 | ARPC2 | C9JTV5 | 2.22 |
|  | CAV1 | C9JKI3 | 0.81 | PRDX4 | A6NG45 | 2.22 |
|  | CD9 | A0A087WU13 | 0.81 | PLOD1 | Q02809 | 2.22 |
|  | CCS | E9PK03 | 0.81 | PFKP | B1APP6 | 2.22 |
|  | CFB | A0A0G2JH38 | 0.81 | RPS23 | D6R9I7 | 2.22 |
|  | LOXL2 | E5RFE2 | 0.8 | H2AFZ | P0C0S5 | 2.21 |
|  | KLF5 | Q13887 | 0.8 | ACTN1 | G3V2E8 | 2.21 |
|  | HIST1H2BF | P62807 | 0.8 | PABPC1 | A0A087WTT1 | 2.21 |
|  | TIMP3 | P35625 | 0.8 | ACTN4 | F5GXS2 | 2.21 |
|  | COPA | P53621 | 0.8 | GARS | F8WCK4 | 2.21 |
|  | CLIC1 | O00299 | 0.79 | TCP1 | E7EQR6 | 2.2 |
|  | RHOC | E9PLA2 | 0.79 | CAPN2 | C9JWY7 | 2.2 |
|  | CSNK2B | A0A0G2JM12 | 0.79 | RPL19 | J3KTE4 | 2.19 |
|  | NPM3 | O75607 | 0.79 | C1QBP | A0A0G2JLC0 | 2.19 |
|  | ANXA6 | E5RFF0 | 0.79 | HSPA1A | P0DMV8 | 2.18 |
|  | TUBA4A | C9JDL2 | 0.79 | TMED2 | E7EQ72 | 2.17 |
|  | ATP1B1 | P05026 | 0.79 | UQCRH | A0A087WTF2 | 2.17 |
|  | FBLN1 | A0A0U1RRA4 | 0.79 | DAD1 | A0A0B4J239 | 2.16 |
|  | CALR | K7EJB9 | 0.78 | IARS | A0A0A0MSX9 | 2.15 |
|  | ABCC9 | G3V1N6 | 0.78 | MTHFD1 | F5H2F4 | 2.14 |
|  | HIST1H2BE | P62807 | 0.78 | DDOST | A0A0C4DGS1 | 2.14 |
|  | KRAS | G3V4K2 | 0.77 | ANXA1 | P04083 | 2.14 |
|  | LAMB2 | F5H520 | 0.77 | RPS16 | M0QX76 | 2.14 |
|  | ITGB4 | J3KSH9 | 0.77 | PSMB8 | A0A140T998 | 2.14 |
|  | ANXA8 | A0A075B748 | 0.77 | RPL4 | H3BM89 | 2.13 |
|  | LAMA2 | A0A087WX80 | 0.77 | SRSF9 | H0YIB4 | 2.13 |
|  | HIST1H2AE | P04908 | 0.77 | STRAP | F5H1G1 | 2.13 |
|  | PLOD2 | C9JXZ0 | 0.77 | RPS3 | E9PJH4 | 2.12 |
|  | ACTN1 | G3V2E8 | 0.76 | FTL | A0A087X1B9 | 2.12 |

Supplementary Table 5 Top 100 highly ranked network-based genes in ToppGene.

|  | **Up-regulated Network** | | | | **Down-regulated Network** | | | |
| --- | --- | --- | --- | --- | --- | --- | --- | --- |
| **Rank** | **Gene** | **Uniprot ID** | **Interactant count** | **Score** | **Gene** | **Uniprot ID** | **Interactant count** | **Score** |
|  | FBXO6 | J3KQ72 | 622 | 3.98E-03 | NTRK1 | E9PQG0 | 1954 | 4.18E-03 |
|  | NTRK1 | E9PQG0 | 1954 | 3.49E-03 | APP | A0A0A0MRG2 | 2096 | 3.80E-03 |
|  | ELAVL1 | M0QZR9 | 1768 | 3.29E-03 | ELAVL1 | M0QZR9 | 1768 | 2.88E-03 |
|  | APP | A0A0A0MRG2 | 2096 | 3.08E-03 | EED | E9PJK2 | 1337 | 2.52E-03 |
|  | EED | E9PJK2 | 1337 | 2.59E-03 | CUL3 | A0A087WTG3 | 1209 | 2.36E-03 |
|  | ATF7IP | A8MV73 | 83 | 2.32E-03 | PLXNC1 | B4DHQ7 | 1 | 2.22E-03 |
|  | TJP1 | A0A087X0K9 | 106 | 2.19E-03 | FN1 | H0Y4K8 | 765 | 2.15E-03 |
|  | MCM2 | C9J013 | 908 | 2.05E-03 | MCM2 | C9J013 | 908 | 2.15E-03 |
|  | CUL3 | A0A087WTG3 | 1209 | 1.88E-03 | FBXO6 | J3KQ72 | 622 | 2.04E-03 |
|  | NXF1 | B4E227 | 1119 | 1.81E-03 | NXF1 | B4E227 | 1119 | 2.01E-03 |
|  | XPO1 | C9IYM2 | 1229 | 1.71E-03 | MOV10 | Q5JR04 | 993 | 1.82E-03 |
|  | MOV10 | Q5JR04 | 993 | 1.65E-03 | XPO1 | C9IYM2 | 1229 | 1.75E-03 |
|  | TP53 | A0A087WT22 | 1019 | 1.48E-03 | EGFR | A0A0B4J1Y5 | 878 | 1.69E-03 |
|  | CACNA1A | A0A087WW63 | 103 | 1.48E-03 | CDK2 | E7ESI2 | 669 | 1.66E-03 |
|  | CUL7 | Q14999 | 646 | 1.42E-03 | COPS5 | E5RFS1 | 798 | 1.62E-03 |
|  | COPS5 | E5RFS1 | 798 | 1.41E-03 | TP53 | A0A087WT22 | 1019 | 1.51E-03 |
|  | MYC | A0A087WUS5 | 662 | 1.39E-03 | CUL7 | Q14999 | 646 | 1.46E-03 |
|  | ATXN7 | H0YAT6 | 78 | 1.34E-03 | EWSR1 | A0A0D9SFL3 | 706 | 1.30E-03 |
|  | CAND1 | A0A0C4DGH5 | 693 | 1.32E-03 | ESR1 | B0QYW7 | 757 | 1.28E-03 |
|  | PTPRK | A0A0G2JQF6 | 92 | 1.30E-03 | SIRT7 | I3L2A4 | 663 | 1.26E-03 |
|  | CUL1 | A0A0C4DGX4 | 680 | 1.26E-03 | CUL1 | A0A0C4DGX4 | 680 | 1.25E-03 |
|  | HSP90AA1 | A0A0U1RR69 | 793 | 1.24E-03 | BAG3 | C9JFK9 | 447 | 1.25E-03 |
|  | SIRT7 | I3L2A4 | 663 | 1.23E-03 | MYC | A0A087WUS5 | 662 | 1.20E-03 |
|  | ESR1 | B0QYW7 | 757 | 1.16E-03 | CAND1 | A0A0C4DGH5 | 693 | 1.16E-03 |
|  | RNF2 | Q99496 | 770 | 1.16E-03 | RNF2 | Q99496 | 770 | 1.16E-03 |
|  | CDK2 | E7ESI2 | 669 | 1.15E-03 | ITGA4 | E7EP60 | 512 | 1.14E-03 |
|  | EWSR1 | A0A0D9SFL3 | 706 | 1.13E-03 | GRB2 | J3KT38 | 582 | 1.08E-03 |
|  | CCDC8 | Q9H0W5 | 543 | 1.13E-03 | OBSL1 | A6NN50 | 615 | 1.07E-03 |
|  | HNRNPA1 | F8VTQ5 | 527 | 1.10E-03 | PTN | C9JR52 | 98 | 1.06E-03 |
|  | OBSL1 | A6NN50 | 615 | 1.09E-03 | HUWE1 | A0A087X146 | 461 | 1.04E-03 |
|  | GRB2 | J3KT38 | 582 | 1.04E-03 | VCAM1 | E9PDD2 | 446 | 1.04E-03 |
|  | POU5F1 | A0A087WYB7 | 573 | 1.01E-03 | HSPB1 | C9J3N8 | 401 | 1.03E-03 |
|  | PLG | A6PVI2 | 65 | 9.79E-04 | HECW2 | C9JHL2 | 290 | 9.88E-04 |
|  | ITGA4 | E7EP60 | 512 | 9.46E-04 | PARK2 | A0A087WU39 | 421 | 9.83E-04 |
|  | HSPA5 | P11021 | 425 | 9.19E-04 | CCDC8 | Q9H0W5 | 543 | 9.68E-04 |
|  | VCP | C9IZA5 | 605 | 9.12E-04 | IQCB1 | C9J6Z7 | 295 | 9.13E-04 |
|  | TAZ | A0A087WWD5 | 93 | 9.10E-04 | NPM1 | E5RGW4 | 593 | 9.07E-04 |
|  | BRCA1 | A0A0A0MSN1 | 543 | 9.02E-04 | TERF1 | E5RFJ5 | 310 | 9.03E-04 |
|  | KRAS | G3V4K2 | 443 | 8.93E-04 | YWHAQ | E9PG15 | 445 | 8.87E-04 |
|  | CUL5 | E9PP19 | 428 | 8.76E-04 | KCNMA1 | A0A087WZL8 | 185 | 8.61E-04 |
|  | CDC5L | Q99459 | 582 | 8.64E-04 | POU5F1 | A0A087WYB7 | 573 | 8.48E-04 |
|  | VCAM1 | E9PDD2 | 446 | 8.55E-04 | CDC5L | Q99459 | 582 | 8.48E-04 |
|  | TMEM17 | Q86X19 | 400 | 8.38E-04 | UBE2I | B0QYN7 | 452 | 8.45E-04 |
|  | UCHL5 | H0Y4E0 | 213 | 8.15E-04 | CUL2 | A0A0A0MTN0 | 451 | 8.41E-04 |
|  | TERF1 | E5RFJ5 | 310 | 8.10E-04 | FUS | H3BNZ4 | 327 | 8.20E-04 |
|  | ACTB | C9JTX5 | 319 | 8.03E-04 | PSMA3 | G3V3W4 | 275 | 8.07E-04 |
|  | HNRNPU | Q00839 | 517 | 8.01E-04 | HDAC5 | K7EJL4 | 350 | 8.03E-04 |
|  | TMEM25 | E9PI73 | 77 | 7.97E-04 | CSNK2A1 | A0A087WY74 | 473 | 7.78E-04 |
|  | SOX2 | P48431 | 362 | 7.84E-04 | BRCA1 | A0A0A0MSN1 | 543 | 7.74E-04 |
|  | PTCH1 | A0A0C4DGI4 | 94 | 7.75E-04 | SOD1 | H7BYH4 | 154 | 7.71E-04 |
|  | SIAE | Q9HAT2 | 50 | 7.75E-04 | CUL5 | E9PP19 | 428 | 7.71E-04 |
|  | YWHAZ | B0AZS6 | 515 | 7.63E-04 | RPA2 | P15927 | 442 | 7.55E-04 |
|  | EP300 | A0A0U1RQG3 | 500 | 7.63E-04 | MDM2 | A0A0C4DFR5 | 419 | 7.54E-04 |
|  | SNW1 | G3V3A4 | 527 | 7.53E-04 | UBC | F5GXK7 | 431 | 7.42E-04 |
|  | BAG3 | C9JFK9 | 447 | 7.53E-04 | HDAC1 | Q13547 | 550 | 7.40E-04 |
|  | HDAC1 | Q13547 | 550 | 7.45E-04 | SNW1 | G3V3A4 | 527 | 7.38E-04 |
|  | HUWE1 | A0A087X146 | 461 | 7.34E-04 | SHMT2 | G3V241 | 428 | 7.38E-04 |
|  | CUL2 | A0A0A0MTN0 | 451 | 7.33E-04 | EEF1A1 | A0A087WV01 | 386 | 7.36E-04 |
|  | RPA1 | I3L2M5 | 469 | 7.29E-04 | HNRNPU | Q00839 | 517 | 7.34E-04 |
|  | RPA2 | P15927 | 442 | 7.24E-04 | RPA1 | I3L2M5 | 469 | 7.23E-04 |
|  | YWHAE | B4DJF2 | 481 | 7.14E-04 | EP300 | A0A0U1RQG3 | 500 | 7.16E-04 |
|  | MCM5 | B1AHA9 | 232 | 7.00E-04 | ARRB2 | I3L0V6 | 352 | 7.03E-04 |
|  | SRC | P12931 | 348 | 6.97E-04 | NOTCH2NL | Q7Z3S9 | 244 | 7.00E-04 |
|  | CUL4B | A6NE76 | 342 | 6.97E-04 | HSP90AB1 | P08238 | 316 | 6.91E-04 |
|  | YWHAQ | E9PG15 | 445 | 6.95E-04 | PAN2 | F8VXK8 | 343 | 6.72E-04 |
|  | PDIA3 | F8WBS6 | 119 | 6.92E-04 | SMAD4 | A0A087WUF3 | 253 | 6.66E-04 |
|  | MYH9 | B1AH99 | 318 | 6.83E-04 | CUL4B | A6NE76 | 342 | 6.45E-04 |
|  | SKIL | C9J8R9 | 106 | 6.79E-04 | TMEM17 | Q86X19 | 400 | 6.44E-04 |
|  | UBE2I | B0QYN7 | 452 | 6.61E-04 | SRPK2 | C9J2M4 | 449 | 6.41E-04 |
|  | TUBG1 | K7EIS0 | 236 | 6.57E-04 | PTEN | A0A087WT17 | 268 | 6.39E-04 |
|  | PARK2 | A0A087WU39 | 421 | 6.51E-04 | PCNA | P12004 | 293 | 6.37E-04 |
|  | SMURF2 | J3QLG1 | 122 | 6.49E-04 | NANOG | F5GZI2 | 476 | 6.33E-04 |
|  | TINF2 | H0YKA6 | 111 | 6.48E-04 | VHL | P40337 | 298 | 6.27E-04 |
|  | HSPA8 | A8K7Q2 | 397 | 6.48E-04 | XIAP | B1AKU2 | 143 | 6.27E-04 |
|  | PPP1CC | A0A087WYY5 | 292 | 6.47E-04 | HSPE1 | B8ZZ54 | 118 | 6.25E-04 |
|  | H2AFX | P16104 | 223 | 6.47E-04 | MEOX2 | P50222 | 214 | 6.24E-04 |
|  | TRAF6 | Q9Y4K3 | 300 | 6.46E-04 | ACTB | C9JTX5 | 319 | 6.23E-04 |
|  | PAN2 | F8VXK8 | 343 | 6.46E-04 | H2AFX | P16104 | 223 | 6.20E-04 |
|  | NANOG | F5GZI2 | 476 | 6.45E-04 | KRAS | G3V4K2 | 443 | 6.20E-04 |
|  | COL1A1 | I3L3H7 | 57 | 6.27E-04 | SUZ12 | J3QQW9 | 344 | 6.15E-04 |
|  | IQCB1 | C9J6Z7 | 295 | 6.22E-04 | TAGLN2 | P37802 | 112 | 6.09E-04 |
|  | EEF1A1 | A0A087WV01 | 386 | 6.21E-04 | UBL4A | F8WB70 | 297 | 6.04E-04 |
|  | SRPK2 | C9J2M4 | 449 | 6.16E-04 | AKT1 | A0A087WY56 | 323 | 6.04E-04 |
|  | HSPB1 | C9J3N8 | 401 | 6.15E-04 | ATF2 | B8ZZU6 | 215 | 6.04E-04 |
|  | TCTN3 | A0A0C4DFN5 | 316 | 6.14E-04 | RPA3 | B5MC59 | 380 | 6.02E-04 |
|  | LGALS9 | J3KS82 | 72 | 6.14E-04 | MSN | P26038 | 126 | 6.00E-04 |
|  | NEDD8 | F8VSA6 | 260 | 6.14E-04 | NEDD8 | F8VSA6 | 260 | 5.99E-04 |
|  | COL4A2 | A0A087WY39 | 41 | 6.11E-04 | UNK | K7EIZ1 | 295 | 5.96E-04 |
|  | SHMT2 | G3V241 | 428 | 6.04E-04 | UBQLN4 | Q9NRR5 | 185 | 5.94E-04 |
|  | RPA3 | B5MC59 | 380 | 6.00E-04 | SRC | P12931 | 348 | 5.91E-04 |
|  | PRKCA | J3KN97 | 259 | 5.98E-04 | POT1 | A8MTK3 | 211 | 5.91E-04 |
|  | SMAD3 | H0YKE2 | 372 | 5.98E-04 | CTNNB1 | B4DGU4 | 349 | 5.80E-04 |
|  | HECW2 | C9JHL2 | 290 | 5.95E-04 | FLNA | A0A087WWY3 | 298 | 5.78E-04 |
|  | HSPA4 | A0A087WTS8 | 320 | 5.93E-04 | BMI1 | P35226 | 370 | 5.78E-04 |
|  | HSP90AB1 | P08238 | 316 | 5.93E-04 | TCTN3 | A0A0C4DFN5 | 316 | 5.77E-04 |
|  | UBC | F5GXK7 | 431 | 5.93E-04 | SMURF1 | Q9HCE7 | 314 | 5.67E-04 |
|  | SFN | P31947 | 263 | 5.93E-04 | AURKA | A3KFJ0 | 273 | 5.66E-04 |
|  | CUL4A | A0A087WWN2 | 279 | 5.90E-04 | FBXW11 | E5RG78 | 376 | 5.55E-04 |
|  | HDAC5 | K7EJL4 | 350 | 5.87E-04 | UCHL5 | H0Y4E0 | 213 | 5.50E-04 |
|  | XRCC5 | C9JZ81 | 227 | 5.86E-04 | ABCE1 | D6R9I9 | 238 | 5.45E-04 |

**Supplementary** **Table 6 Comparison between the three top highly ranked proteins in up-regulated and down-regulated protein datasets.**

| **Up-regulated Network** | | | | | **Down-regulated Network** | | | | |
| --- | --- | --- | --- | --- | --- | --- | --- | --- | --- |
| **Tool^a^** | **Gene Name** | **Gene ID** | **Uniprot ID** | **Protein name** | **Tool^a^** | **Gene Name** | **Gene ID** | **Uniprot ID** | **Protein name** |
| N,G | ANXA1 | 301 | P04083 | Annexin A1 | N,G | PSMB3 | 5691 | P49720 | Proteasome subunit beta 3 |
| N,G | ANXA7 | 310 | P20073 | Annexin A7 | N,G | VIM | 7431 | P08670 | Vimentin |
| N,G | LAMA5 | 3911 | O15230 | Laminin subunit alpha 5 | N,G | CSNK2B | 1460 | P67870 | Casein kinase 2 beta |
| N,G | MMP2 | 4313 | P08253 | Matrix metallopeptidase 2 | N,G | NCL | 4691 | P19338 | Nucleolin |
| N,G | ANXA8L2 | 728113 | Q5VT79 | Annexin A8-like 1 | N,G | VDAC1 | 7416 | P21796 | Voltage dependent anion channel 1 |
| N,G | CAV1 | 857 | Q03135 | Caveolin 1 | N,G | PABPC1 | 26986 | P11940 | Poly(A) binding protein cytoplasmic 1 |
| N,G | CSNK2B | 1460 | P67870 | Casein kinase 2 beta | N,T | APP | 351 | P05067 | Amyloid beta precursor protein |
| N,T | FBXO6 | 26270 | Q9NRD1 | F-box protein 6 | N,T | ELAVL1 | 1994 | Q15717 | ELAV like RNA binding protein 1 |
| N,T | ELAVL1 | 1994 | Q15717 | ELAV like RNA binding protein 1 | N,T | FN1 | 2335 | P02751 | Fibronectin 1 |
| N,T | ATF7IP | 55729 | Q6VMQ6 | Activating transcription factor 7 interacting protein | N,T | COPS5 | 10987 | Q92905 | COP9 signalosome subunit 5 |
| N,T | TJP1 | 7082 | Q07157 | Tight junction protein 1 | N,T | GRB2 | 2885 | P62993 | Growth factor receptor bound protein 2 |
| N,T | MYC | 4609 | P01106 | V-myc avian myelocytomatosis viral oncogene homolog | N,T | IQCB1 | 9657 | Q15051 | IQ motif containing B1 |
| N,T | GRB2 | 2885 | P62993 | Growth factor receptor bound protein 2 | N,T | NPM1 | 4869 | P06748 | Nucleophosmin |
| N,T | UCHL5 | 51377 | Q9Y5K5 | Ubiquitin C-terminal hydrolase L5 | N,T | CDC5L | 988 | Q99459 | Cell division cycle 5 like |
| N,T | TERF1 | 7013 | P54274 | Telomeric repeat binding factor 1 | N,T | EP300 | 2033 | Q09472 | E1A binding protein p300 |
| N,T | YWHAZ | 7534 | P63104 | Tyrosine 3-monooxygenase/tryptophan 5-monooxygenase activation protein zeta | N,T | PCNA | 5111 | P12004 | Proliferating cell nuclear antigen |
| N,T | SRC | 6714 | P12931 | SRC proto-oncogene, non-receptor tyrosine kinase | N,T | VHL | 7428 | P40337 | Von Hippel-Lindau tumor suppressor |
| N,T | SKIL | 6498 | P12757 | SKI like proto-oncogene | N,T | H2AFX | 3014 | P16104 | H2A histone family member X |
| N,T | SMURF2 | 64750 | Q9HAU4 | SMAD specific E3 ubiquitin protein ligase 2 | N,T | UBQLN4 | 56893 | Q9NRR5 | Ubiquilin 4 |
| N,T | TRAF6 | 7189 | Q9Y4K3 | TNF receptor associated factor 6 | G,T | VCAM1 | 7412 | P19320 | Vascular cell adhesion molecule 1 |
| N,T | HSPA4 | 3308 | P34932 | Heat shock protein family A (Hsp70) member 4 | G,T | ACTB | 60 | P60709 | Actin beta |
| N,T | UBC | 7316 | P0CG48 | Ubiquitin C | G,T | HSP90AB1 | 3326 | P08238 | Heat shock protein 90 alpha family class B member 1 |
| G,T | KRAS | 3845 | P01116 | KRAS proto-oncogene, GTPase | G,T | HSPE1 | 3336 | P61604 | Heat shock protein family E (Hsp10) member 1 |
| G,T | VCAM1 | 7412 | P19320 | Vascular cell adhesion molecule 1 | N,G,T | PSMA3 | 5684 | P25788 | Proteasome subunit alpha 3 |
| G,T | COL4A2 | 1284 | P08572 | Collagen type IV alpha 2 chain | N,G,T | YWHAQ | 10971 | P27348 | Tyrosine 3-monooxygenase |
| N,G,T | COL1A1 | 1277 | P02452 | Collagen type I alpha 1 chain | N,G,T | UBC | 7316 | P0CG48 | Ubiquitin C |
| N,G,T | XRCC5 | 7520 | P13010 | X-ray repair cross complementing 5 | N,G,T | MSN | 4478 | P26038 | Moesin |

**^a^Abbreviations: N = NetworkAnalyst; G = GeneMANIA; T = ToppGene**

**References**

Bendtsen, J.D., Jensen, L.J., Blom, N., Von Heijne, G., and Brunak, S. (2004). Feature-based prediction of non-classical and leaderless protein secretion. *Protein Eng Des Sel* 17**,** 349-356.

Chen, J., Bardes, E.E., Aronow, B.J., and Jegga, A.G. (2009). ToppGene Suite for gene list enrichment analysis and candidate gene prioritization. *Nucleic Acids Res* 37**,** W305-311.

Heberle, H., Meirelles, G.V., Da Silva, F.R., Telles, G.P., and Minghim, R. (2015). InteractiVenn: a web-based tool for the analysis of sets through Venn diagrams. *BMC Bioinformatics* 16**,** 169.

Horton, P., Park, K.J., Obayashi, T., Fujita, N., Harada, H., Adams-Collier, C.J., and Nakai, K. (2007). WoLF PSORT: protein localization predictor. *Nucleic Acids Res* 35**,** W585-587.

Huang Da, W., Sherman, B.T., and Lempicki, R.A. (2009). Systematic and integrative analysis of large gene lists using DAVID bioinformatics resources. *Nat Protoc* 4**,** 44-57.

Kall, L., Krogh, A., and Sonnhammer, E.L. (2004). A combined transmembrane topology and signal peptide prediction method. *J Mol Biol* 338**,** 1027-1036.

Kanehisa, M., Sato, Y., Kawashima, M., Furumichi, M., and Tanabe, M. (2016). KEGG as a reference resource for gene and protein annotation. *Nucleic Acids Res* 44**,** D457-462.

Krzywinski, M., Schein, J., Birol, I., Connors, J., Gascoyne, R., Horsman, D., Jones, S.J., and Marra, M.A. (2009). Circos: an information aesthetic for comparative genomics. *Genome Res* 19**,** 1639-1645.

Molloy, K., Smith, S.G., Cagney, G., Dillon, E.T., Greene, C.M., and Mcelvaney, N.G. (2019). Characterisation of the Major Extracellular Proteases of Stenotrophomonas maltophilia and Their Effects on Pulmonary Antiproteases. *Pathogens* 8.

Newman, M.E. (2006). Modularity and community structure in networks. *Proc Natl Acad Sci U S A* 103**,** 8577-8582.

Petersen, T.N., Brunak, S., Von Heijne, G., and Nielsen, H. (2011). SignalP 4.0: discriminating signal peptides from transmembrane regions. *Nat Methods* 8**,** 785-786.

Sonnhammer, E.L., Von Heijne, G., and Krogh, A. (1998). A hidden Markov model for predicting transmembrane helices in protein sequences. *Proc Int Conf Intell Syst Mol Biol* 6**,** 175-182.

Szklarczyk, D., Franceschini, A., Wyder, S., Forslund, K., Heller, D., Huerta-Cepas, J., Simonovic, M., Roth, A., Santos, A., Tsafou, K.P., Kuhn, M., Bork, P., Jensen, L.J., and Von Mering, C. (2015). STRING v10: protein-protein interaction networks, integrated over the tree of life. *Nucleic Acids Res* 43**,** D447-452.

Warde-Farley, D., Donaldson, S.L., Comes, O., Zuberi, K., Badrawi, R., Chao, P., Franz, M., Grouios, C., Kazi, F., Lopes, C.T., Maitland, A., Mostafavi, S., Montojo, J., Shao, Q., Wright, G., Bader, G.D., and Morris, Q. (2010). The GeneMANIA prediction server: biological network integration for gene prioritization and predicting gene function. *Nucleic Acids Res* 38**,** W214-220.

Xia, J., Benner, M.J., and Hancock, R.E. (2014). NetworkAnalyst--integrative approaches for protein-protein interaction network analysis and visual exploration. *Nucleic Acids Res* 42**,** W167-174.

Yang, C., Chen, P., Zhang, W., and Du, H. (2016). Bioinformatics-Driven New Immune Target Discovery in Disease. *Scand J Immunol* 84**,** 130-136.
